# Natural Products for Acetaminophen-Induced Acute Liver Injury: A Review

**DOI:** 10.3390/molecules28237901

**Published:** 2023-12-01

**Authors:** Xiaoyangzi Li, Ruyang Lao, Jiawei Lei, Yuting Chen, Qi Zhou, Ting Wang, Yingpeng Tong

**Affiliations:** 1 School of Medicine, Taizhou University, Taizhou 318000, China; 2137240063@stu.tzc.edu.cn (X.L.); 2037240068@stu.tzc.edu.cn (R.L.); 2037270060@stu.tzc.edu.cn (J.L.); 2 College of Pharmacy, Liaoning University of Traditional Chinese Medicine, Dalian 116000, China; chenyuting0423@163.com; 3 School of Pharmacy, Taizhou University, Taizhou 318000, China; zhouqi0313@tzc.edu.cn

**Keywords:** acetaminophen, acute liver injury, natural products, P450 enzymes

## Abstract

The liver plays a vital role in metabolism, synthesis, and detoxification, but it is susceptible to damage from various factors such as viral infections, drug reactions, excessive alcohol consumption, and autoimmune diseases. This susceptibility is particularly problematic for patients requiring medication, as drug-induced liver injury often leads to underestimation, misdiagnosis, and difficulties in treatment. Acetaminophen (APAP) is a widely used and safe drug in therapeutic doses but can cause liver toxicity when taken in excessive amounts. This study aimed to investigate the hepatotoxicity of APAP and explore potential treatment strategies using a mouse model of APAP-induced liver injury. The study involved the evaluation of various natural products for their therapeutic potential. The findings revealed that natural products demonstrated promising hepatoprotective effects, potentially alleviating liver damage and improving liver function through various mechanisms such as oxidative stress and inflammation, which cause changes in signaling pathways. These results underscore the importance of exploring novel treatment options for drug-induced liver injury, suggesting that further research in this area could lead to the development of effective preventive and therapeutic interventions, ultimately benefiting patients with liver injury caused by medicine.

## 1. Introduction

The liver is a crucial organ responsible for essential physiological functions including metabolism, synthesis, and detoxification [1]. Various factors, such as viral infections, drug reactions, excessive alcohol consumption, and autoimmune diseases, can damage the liver. Severe liver damage can lead to hepatitis, cirrhosis, or even liver cancer, which can have serious consequences. Acute liver failure, which has a sudden onset and high mortality rate, is a particularly concerning condition [2]. Among the different types of liver damage, drug-induced liver injury (DILI) poses a significant challenge to patients.

Reports indicate that DILI is the leading cause of acute liver failure (ALF) in the United States, accounting for approximately 60% of cases [3]. The severity of liver damage emphasizes the critical nature of this condition, underlining the need to study DILI models and identify preventive or therapeutic treatments for it.

Acetaminophen (APAP), also known as paracetamol or *N*-acetylaminophenol, is a safe and effective primary medication for fever, pain, and inflammation when taken at proper therapeutic doses [4]. However, being available as an over-the-counter drug, it is prone to excessive use by patients. While acetaminophen is safe and effective at therapeutic doses, an overdose can lead to liver injury and hepatotoxicity [5]. The recommended therapeutic dose for adults is a total dose of 60 mg/kg over 24 h, with a maximum daily limit of 4 g [6]. For children, the dose is 10–15 mg/kg, with a maximum of 60–75 mg/kg [7,8]. Exceeding a daily dose of ≥10 g or ≥200 mg/kg (whichever is less) of acetaminophen can result in fatal liver damage [6]. Hepatotoxicity encompasses liver damage or liver failure caused by exposure to specific natural, environmental toxins or excessive consumption of certain medications. APAP, an analgesic and antipyretic drug, has been established as safe at therapeutic doses, but can cause severe clinical hepatotoxicity at extremely high doses. Currently, there are no specific effective treatments available for DILI, which further emphasizes the critical nature of this condition [9]. Thus, studying DILI models is crucial for the identification of drugs or plant products with preventive or therapeutic effects on DILI.

In the past few decades, plant chemicals have gained attention in the field of life sciences. In 1968, the scholar D Maclean proposed a treatment for acute poisoning caused by APAP overdose in The Lancet journal. The treatment involved the use of diuretics, intravenous hydrocortisone, and anti-histamine drugs to alleviate symptoms and eliminate excess APAP in the body [10]. In 1990, the scholar A Akintonwa proposed the therapeutic effect of Garcinia kola seed extract on drug-induced liver injury of this nature [11]. This marked the first mention in the PubMed database of the therapeutic effects of plant extracts on this type of disease. As more scholars conducted research, it was found that natural products exert certain hepatoprotective effects through oxidative stress, inflammation, apoptosis, and other mechanisms. The research on natural products not only provides insights into their biological mechanisms but also offers potential drugs for treating APAP-induced acute liver injury. To conduct continuous and in-depth research, this study aims to investigate the impact of natural products on the therapeutic effect of APAP hepatotoxicity through animal and cell experiments. Thus, the evaluation of effective animal and cell models is crucial for the success of this endeavor.

This review aims to provide a comprehensive review and discussion of the model methods for acetaminophen (APAP)-induced liver injury, with the aim of facilitating further research on disease models. Given that previous scholars have already summarized the therapeutic effects of phytochemical agents on APAP-induced liver injury up to 2018 [12], our review seeks to summarize and explore the molecular mechanisms of the actin of phytochemical agents in the past five years. Special emphasis is placed on introducing the research progress and potential issues related to natural products.

The review is based on the results of studies from articles and reviews published for almost five years (2019–2023). We have conducted a literature search using publicly available databases (PubMed and Web of Science), using the keywords “phytochemicals”, “plant extracts”, “traditional herbal medicine”, “plants”, “natural products”, “APAP”, “liver injury”, “hepatotoxicity”, “oxidative stress”, “herbal formula”, and “inflammation”.

## 2. Potential Mechanisms of APAP-Induced Acute Liver Injury

**Figure 1:**
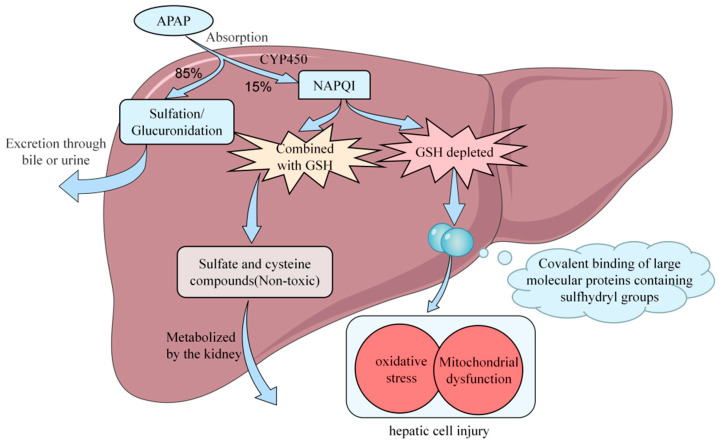
Schematic representation of APAP metabolism in the liver. Upon entering the body, the majority of APAP forms complexes with sulfate and glucuronide, which are then excreted via bile or urine. The remaining portion is metabolized by CYP450 enzymes to form NAPQI, which combines with GSH and is subsequently metabolized by the kidneys. When there is excessive depletion of GSH, NAPQI accumulates in the liver, leading to liver injury. APAP: *N*-acetyl-p-aminophenol; CYP450: cytochrome P450; NAPQI: *N*-acetyl-p-benzoquinone imine; GSH: glutathione. This figure was partly generated using Servier Medical Art, provided by Servier, licensed under a Creative Commons Attribution 3.0 unported license.

Currently, the precise molecular mechanisms underlying APAP-induced acute liver injury are not fully understood. However, most researchers believe that, when therapeutic concentrations of APAP enter the body, approximately 85% of APAP undergoes sulfation/glucuronidation to form complexes that are excreted via bile or urine, while approximately 15% undergoes conversion through the cytochrome P450 (CYP450) pathway. In the human body, APAP is converted to a toxic compound called N-acetyl-p-benzoquinone imine (NAPQI) by an enzyme called P4502E1. However, this toxic compound is quickly detoxified with glutathione to form harmless sulfate and cysteine compounds. The kidneys then metabolize these compounds [13]. P4502A6 selectively metabolizes APAP into the nontoxic catechol metabolite 3-hydroxy-APAP (3-OH-APAP) [14]. During a drug overdose, more APAP is converted by cytochrome P450 enzymes into NAPQI, leading to liver injury through mechanisms including oxidative stress, binding to macromolecules, mitochondrial dysfunction, and endoplasmic reticulum stress [13,14] (Figure 1).

**Figure 2:**
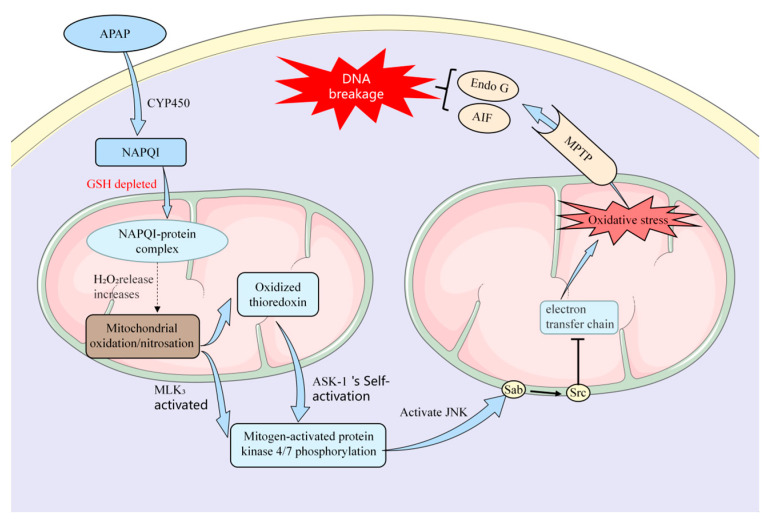
Molecular mechanisms underlying APAP-induced hepatocyte injury and death. APAP in hepatocytes is metabolized by cytochrome P450 into *N*-acetyl-p-benzoquinone imine (NAPQI). High levels of NAPQI deplete GSH stores and form NAPQI–protein complexes, damaging the respiratory chain and enhancing the generation of reactive oxygen species (ROS) such as superoxide. Nitric oxide (NO) within the mitochondria nitrosylates mitochondrial proteins, impairing mitochondrial antioxidant defense function and leading to mitochondrial oxidative stress, as well as the oxidation of proteins such as mitochondrial thioredoxin. Oxidized thioredoxin dissociates from its binding partner ASK1, resulting in the activation of ASK1. Activated ASK1, in conjunction with activated MLK3 via MKK4/7 phosphorylation, activates JNK. Activated JNK translocates to the outer mitochondrial membrane and associates with Sab, leading to the inhibition of mitochondrial electron transfer through Src-mediated signaling, thus amplifying mitochondrial oxidative stress. This leads to a transition in mitochondrial permeability, resulting in the release of intermembrane proteins such as Endo G and AIF. AIF and Endo G translocate to the nucleus, inducing fragmentation of nuclear DNA, ultimately leading to hepatocyte death. APAP: *N*-acetyl-p-aminophenol; NAPQI: *N*-acetyl-p-benzoquinone imine; ASK1: apoptosis signal-regulating kinase 1; MLK3: mixed-lineage kinase 3; MKK4/7: mitogen-activated protein kinase 4/7; Endo G: endonuclease G; AIF: apoptosis-inducing factor; JNK: c-jun *N*-terminal kinase. This figure was partly generated using Servier Medical Art, provided by Servier, licensed under a Creative Commons Attribution 3.0 unported license.

After excessive ingestion of APAP, a small amount of NAPQI can be converted by glutathione (GSH) in the mitochondria into a less harmful reduced form, which is then excreted via bile. However, the accumulated NAPQI covalently binds to macromolecules containing thiol groups and other electron-rich moieties, inhibiting protein activity in liver cells and resulting in liver injury [15]. Furthermore, following the depletion of GSH, the production of reactive oxygen species (ROS) increases concomitantly with the consumption of GSH by NAPQI. This leads to the oxidation of thioredoxin and the dissociation of apoptosis signal-regulating kinase 1 (ASK-1) from the protein, triggering the self-activation of ASK-1. This phosphorylates c-Jun amino-terminal kinase (JNK) through mitogen-activated protein kinase 4/7 (MK4/7), leading to JNK activation [16]. Activated JNK may inhibit Src in the mitochondrial electron transfer chain function and increase ROS production, thereby sustaining JNK activation [17]. This further promotes an increase in ROS, triggering a cascade of free radical reactions, causing oxidative stress, and accelerating cellular lipid peroxidation [18], which leads to the impairment of biomolecular function and ultimately results in liver injury. Additionally, NAPQI and ROS selectively deactivate adenine nucleotide translocase (ATP) in the ATPase complex at high-affinity sites, reducing mitochondrial membrane permeability and inducing mitochondrial damage [19]. Furthermore, p-JNK activates Bax, which translocates to the mitochondria, resulting in the mitochondrial permeability transition (MPT) pore opening [20]. The depletion of ATP and the collapse of the membrane potential ultimately lead to DNA fragmentation and cell death. The mechanism of APAP-induced endoplasmic reticulum (ER) stress is still under investigation. The underlying reasons for this occurrence could be attributed to alterations in peroxisomal homeostasis induced by NAPQI, as well as excessive ROS generation and mitochondrial dysfunction. ER stress may exacerbate damage or render liver cells more sensitive to other injuries. A study (Figure 2) showed that the toxicity of excessive APAP ingestion is mainly due to the excessive formation of NAPQI resulting from CYP metabolism [21].

## 3. Models of APAP-Induced Acute Liver Injury

The preparation of the APAP model plays a crucial role in studying its mechanism. However, the current treatment drugs still have limitations, and there is a need for better drugs to study this phenomenon. Theoretical understanding suggests that the preparation of models is critical.

This study aims to evaluate the reliability and validity of various modeling methods for APAP-induced acute liver injury in both in vivo (animal) and in vitro (cellular) models. Additionally, the study will discuss the benefits, limitations, and potential applications of these methods. By summarizing the search for more appropriate modeling techniques, this study hopes to help researchers choose suitable methods for their future experiments, promote the exploration of the underlying mechanisms of the disease, and facilitate the discovery and testing of drugs with relevant therapeutic molecular mechanisms. Ultimately, the findings of this study may provide a new basis for the successful preparation of APAP-induced acute liver injury models and the intervention of drugs in these models.

### 3.1. In Vivo Models

When there is a need to study the toxicological properties of APAP, small animal models are required. These models should allow researchers to study the drug on a relatively large sample size within a relatively short period of time. Additionally, they should enable researchers to evaluate the extent of liver damage and the efficacy of potential liver protection therapies. Among the animal models commonly used that meet these criteria are laboratory mice and rats.

We found that small animal models have been widely used to simulate APAP-induced liver injury (Table 1). The majority of scholars have used mice as in the models, while a few used rats. The APAP dose used in mouse models is often between 300–500 mg/kg, while, in rat models, it is between 1–2 g/kg. Early studies showed that mice can rapidly develop APAP hepatotoxicity after a single dose, and the mechanism is similar to that in humans [22]. On the other hand, rats seem to have high resistance to APAP-induced hepatotoxicity, and the mechanism of liver injury is not closely related to mitochondrial dysfunction and oxidative stress [22]. Therefore, mice are the most commonly used model for studying APAP-induced liver injury, and they provide assistance for subsequent clinical studies.

There is still some controversy regarding fasting in experimental animals before APAP administration. According to the literature, approximately 46.9% of experiments involved fasting the animals for 12–24 h before APAP administration. Fasting can reduce the influence of diet on liver GSH. However, many scholars believe that fasting is not essential. Fasting may induce autophagy, which can selectively clear damaged mitochondria and APAP-AD to prevent or alleviate liver injury [23], which may interfere with the study of drug mechanisms and efficacy in liver injury.

There are some differences in the administration methods in mice and rats. Mice are often administered the drug intraperitoneally (i.p.), while rats are administered the drug orally (p.o.) or by gavage (i.g.). When administering APAP to mice, factors such as absorption efficiency and controllable variables need to be considered to ensure its quick absorption. However, due to the larger size of rats, injection control is enhanced, making it more appropriate to simulate the oral administration route, which is similar to that used usually.

In the past 50 years, the major serum biomarkers used for screening and monitoring DILI include alanine transaminase (ALT), aspartate transaminase (AST) [24], alkaline phosphatase (ALP) [25], and total bilirubin (TBIL). The level of serum ALT is commonly used as a reliable biochemical marker to assess early hepatic injury in medical evaluations. The primary characteristics of hepatocellular liver injury include hepatocyte necrosis, the infiltration of lymphocytes and eosinophils in the liver, mild bile stasis, an inflammatory response, a significant increase in serum levels of AST and ALT, and moderate increases in levels of gamma-glutamyl transferase (GGT) and ALP [26]. ALP can serve as a specific indicator for cholestatic liver injury and severe DILI. TBIL is a more direct reflection of liver function compared to ALT, AST, and ALP, making it an important parameter for the staging and prognostic evaluation of DILI. However, these serum biomarkers cannot specifically identify DILI and do not indicate potential DILI before clinical liver injury occurs during drug therapy [27].

**Table 1:** Summary of animal model methods.

Sex, Strain, Age/Body, Weight	Dose of APAP	Pre Administration or Treatment	Administration Frequency	Administration	Experimental Index	No. Reference
Male C57BL/6J mice 8–10 weeks	300, 500 mg/kg	Fasted overnight	Once	i.p.	Protein (Pro-CRAMP, CRAMP, CD11b, CYP2E1, JNK, p-JNK, Cyclin D1), Serum Index (ALT), Histology, Tissues (ROS, GSH), Cell quantification (Ki67 cells, neutrophils and macrophages)	Zhai, et al., 2023 [28]
Male C57BL/6J mice 6–8 weeks	300, 500 mg/kg	Fasted for 15–17 h	Once	i.p.	Serum Index (ALT, AST, TNF-α, IL-6, MCP-1, mtDNA), Histology, Tissues (MDA, Caspase-3), Protein (P2RX1, BCL-2, BCL-X, STING, p-STING, TBK1, p-TBK1, P65, p-P65), RNA (*P2RX1*, *TNF-α*, *IL-6*, *MCP-1*)	Yu, et al., 2023 [29]
Male C57BL/6J mice 6–8 weeks	250, 450, 550 mg/kg	Fasted for 16 h	Once	i.p.	Serum Index (ALT, AST, cfDNA, HMGB1, TNF-α, MCP-1), Histology, Cell quantification (leukocytes, neutrophils), Tissues (MDA, GSH-Px), RNA (*Tnf*, *Il1β*, *Mmp3, Sphk1*, *Alox12*, and *Nqo1*, *TNF-α*, *TGF-β*, *3-NT*)	Sun, et al., 2023 [30]
Male C57BL/6N mice 4–8 weeks	300, 600 mg/kg	Fasted overnight	Once	i.p.	Serum Index (ALT, AST, LDH, HMGB1), Protein (NEDD4-1, P-JNK, JNK, Bax, CYPD, VDAC1, COX IV, AIF, Endo G, Cyt C), RNA (*NEDD4-1*, *Vdac1*), Histology, Gene sets, Tissues (ROS, ATP)	Zhu, et al., 2023 [31]
Male C57BL/6N mice 6–8 weeks 18–22 g	250 mg/kg	Fasted for 12 h but free water	Once	i.p.	Serum Index (ALT, AST), Tissues (GSH), mRNA (*cGAS*, *STING*, *IFN-β1*), Protein (ST2)	Wang, et al., 2023 [32]
Male C57BL/6 mice 5–7 weeks 18–22 g	500 mg/kg	Fasted overnight	Once	i.p.	Histology (mitochondria), Protein (γH2AX, p-JNK, JNK, p-Src, Src, p-ATM, ATM, γH2AX, H2AX, p21), Serum Index (ALT, AST, GSH), Tissues (ATP, ROS)	Cen, et al., 2023 [33]
Male C57BL/6 mice 8 weeks	300 mg/kg	-	Once	p.o. for 24 h	Plasma Index (ALT, AST, TNF-α, IL-6, MCP-1, MCP-3, mtDNA), Tissues (SOD, CAT, GSSG, GSH, GSH-Px, MDA, NAPQI, ROS, Fe^2+^), DA, β-galactosidase, β-glucosidase, LDH, Histology, *Lactobacillus* species, Cell quantification (cell death, neutrophils, macrophages), Protein (CYP2E1, CYP1A2, PCNA, p-ASK, p-MKK4, p-JNK, GPX4, xCT), mRNA (*Ptgs2*, *Fdps*)	Zeng, et al., 2023 [34]
Male C57BL/6J mice 8 weeks	200, 400, 600 mg/kg	Fasted for 16 h but free water	Once	i.p.	Plasma Index (ALT, AST, miR122), Histology, mRNA (*Cytochrome P450, HNF1AOS1*), Enzyme Activities (CYP3A11, 1A2, 2B10, 2C29, 2E1), Sedation time	Bao, et al., 2022 [35]
Male C57BL/6 mice 8–9 weeks 18–22g	300 mg/kg	Fasted overnight	Once	i.p.	Plasma Index (ALT, AST, ALB, TBIL), Histology, Tissues (MPO), mRNA (*CCR 2*, *CCR 5*, *CXCL 9*, *CXCR 2*, *IL-1β*, *IL-6*, *TNF-α*, *CAT*, *GSH-PX*, *T-SOD*), Protein (p-JAK2, JAK2, p-STAT3, STAT3, BAX, BCL-2, p-p65, p65)	He, et al., 2022 [36]
Male C57BL/6J mice 8–10 weeks	300 mg/kg	Fasted overnight	Once	i.p.	Serum Index (ALT, MANF), Macrophages, Neutrophils, Histology, mRNA (*Tnfa*, *Il1b*, *Il6*, *Ifna*, *Il10*), Protein (p-AKT, AKT, p-JNK, JNK, p-p38, p-38, p-ERK, ERK, p-SAPK, SAPK), RNA (*Gpnmb*, *Axl*, *Cd36*, *Cd5l*, *Macro*, *Mertk*, *Cd81*, *Trem2*, *CD36*, *CD64*, *THBS1*, *SIRPA*, *MSR1*)	Hou, et al., 2022 [37]
C57BL/6J mice 6–8 weeks	300, 750 mg/kg	Fasted for 12 h	Once	i.p.	Serum Index (ALT, AST, LDH, CK18-M30, CK18-M65, EGR1), Histology, Tissues (GSH, TG), mRNA (*Egr1*, *Acaa2*), Protein (Egr1, CYP2E1, HSP90)	Lei, et al., 2022 [38]
Male C57BL/6N mice 18–28 g	250 mg/kg	Fasted for 12 h	Once	i.p.	Serum Index (ALT, AST, GSH, TNF-α, IL-6, IL-10), Histology, Protein (IL-33, IL-6, IL-1β, AMPKα, p-AMPKα, PI3K, p-PI3K, MEK, ERK, p-ERK, Akt, p-Akt, Beclin-1, LC3I, LC3II), mRNA (*IL-33*, *ST2*, *Cyp1A2*, *Cyp2E1*, *TNF-α*, *IL-6*, *IL-10*, *iNOS*, *IL-12*, *Arg-1*, *IL-10*)	Wang, et al., 2021 [39]
Male C57BL/6J mice 2–3 months	500 mg/kg	-	Once	Feed or i.p.	Serum Index (ALT, VWF), Histology, Protein (p62, LC3II, CYP2E1, APAP-AD, p-p62, GCLC, GCLM, p-JNK, T-JNK, S6, p-S6, 4EBP1, p-4EBP1, Cyclin D1, PCNA, P21), mRNA (*p62*/*Sqstm1*, *Atg8*/*LC3B*, *Gclc*, *Gclm*), Tissues (GSH, APAP-Cys)	Qian, et al., 2021 [40]
Male and female C57BL/6J mice 8–12 weeks	210 mg/kg (male) 325 mg/kg (female)	Fasted overnight	Once	i.p.	Serum Index (ALT, Chi3l1), Histology, Protein (CYP2E1, Chi3l1, CD44, His, NAPQI), Tissues (GSH), Mϕs, Platelet	Shan, et al., 2021 [41]
Male C57BL/6 mice 10 weeks	300 mg/kg	Fasted for 24 h	Once	i.g.	Serum Index (ALT, AST, IL-1β, IL-18, LDH), Histology, Protein (PRX3, NLRP3, GSDMD, Caspase-1, Cleaved Caspase-1, IL-1β, IL-18, PRX5, PRX6), mRNA (*PRX3*)	Wang, et al., 2021 [42]
Male C57BL/6 mice 8–12 weeks	250 mg/kg	Fasted overnight for 12 h	Once	i.p.	Plasma Index (ALT, sPD-L1, AST, TNF-α, IL-6, IL-10), Histology, Cell quantification (Macrophages, KCs, *E. coli*), Protein (PD-1, PD-L1), mRNA (*Pdl1*)	Triantafyllou, et al., 2021 [43]
Male C57BL/6 mice 6–8 weeks 19–20 g	400 mg/kg	Free food and water	Once	i.p.	Histology, Serum Index (ALT, AST), Protein (Total SIRT6, Nuclear SIRT6, SIRT1-7)	Zhou, et al., 2021 [44]
Male C57BL/6 mice	350 mg/kg	-	Once	i.p.	Histology, Serum Index (ALT, AST), Tissues (GSH, MDA, ROS), Oil Red O, Protein (SIRT1, GPX4, NRF2, HO-1)	Wang, et al., 2021 [45]
Male Kunming mice 20–25 g	400 mg/kg	Fasted overnight	Once	i.p.	Serum Index (ALT, AST, IL-1β, TNF-α, IL-6), Tissues (SOD, CAT, GSH, MDA), Histology, Protein (Nrf2, NQO1, HO-1, p-p38, p38, p-p65, p65, iNOS, Bcl-2, Bax, Caspase-3, Caspase-9)	Wang, et al., 2021 [46]
Male and female C57BL/6J-Tg mice 3 months	500 mg/kg	-	Once	i.p.	Serum Index (ALT, AST), Histology, Mitochondrial function assay, Lysophosphatidylcholine, iPLA2, Protein (PRDX6, PRDX6-SO_3_, JNK, pJNK, Bax, Bcl-2)	Lee, et al., 2020 [47]
Male C57BL/6 J mice 8–10 weeks	200, 250, 300, 700 mg/kg	Fasted overnight for 12 h	Once	i.p.	Serum Index (ALT, AST, TNF-α, IL-1β, IL-6), Histology, Protein (SPHK1, SPHK2, IRE1α, STAT1, p65, CYP2E1, CHOP, p-IRE1α, IRE1α, PERK, p-PERK, p-elF2α, elF2α, ATF4, ATF6, p-JNK, JNK, p-ASK1, ASK1, p-GSK3β, GSK3β, p38, TRAF2), Tissues (GSH), Cell quantification (neutrophils, macrophages), mRNA (*TNF-α*, *IL-1β*, *IL-6*, *Ccl2*, *Ccl3*, *Cxcl1*, *Cxcl2*)	Li, et al., 2020 [48]
Male C57BL/6J mice 6–8 weeks	300 mg/kg	Fasted overnight, but free water	Once	i.p.	Serum Index (ALT), Protein (Nqo1, AKR1C, Gstα3, Gstm1, Gstm5, Nrf2, p-JNK, JNK), mRNA (*NQO1, AKR1C*), Histology	Chen, et al., 2020 [49]
Male and female mice 8–10 weeks	210 mg/kg (male) 325 mg/kg (female)	Fasted overnight for 18 h	Once	i.p.	Histology, Serum Index (ALT, IL-6, TGF-β, IL-13), Tissues (GSH, TNF-α, IL-1β, IL-4, IL-10, TGF-β, IL-13), Cell quantification (macrophages, neutrophils), Protein (HIF-2α, CYP2E1), mRNA (*PAI-1*, *ADM*, *VEGF*, *IL-6*, *IL-1β*, *IL-4*, *IL-10*, *MCP-1*, *IFN-γ*, *TNF-α*)	Gao, et al., 2020 [50]
Male mice 8–10 weeks	300, 500 mg/kg	Fasted overnight	Once	i.p.	Histology, Serum Index (ALT, AST, CCL5), mRNA (*Ccl5*, *CD206*, *Ym1*, *Arg1*, *iNOS*, *IL-1β*, *TNF-α*), Tissues (GSH), Protein (PCNA, CD206, Ym1, Arg1, p-Erk1/2, Erk1/2, p-JNK, JNK, p-NF-κB, NF-κB, IκBα, p-IκB, CCR1, CCR5)	Li, et al., 2020 [51]
Male mice 8–10 weeks	300, 500 mg/kg, 750 mg/kg (No fasted)	Fasted for 15–17 h	Once	i.p.	Histology, Serum Index (ALT, AST, TNF-α, IL-6), Protein (OPN, JNK, p-JNK, CYP2E1), Tissues (MDA), Ketone, mtDNA, Cell quantification (neutrophils)	Wen, et al., 2019 [52]
Male C57BL/6J mice 8–12 weeks	500 mg/kg	Free food and water	Once	i.p.	Serum Index (ALT), Histology, Tissues (GSH, APAP-protein adducts), mitochondria bioenergetics, Protein (Ub, p62, Mfn1, Tom20)	Wang, et al., 2019 [53]
C57BL/6J mice	250, 500 mg/kg	Fasted overnight	Once	i.p.	Serum Index (ALT, AST), Protein (p-JNK, JNK, PUMA, Endo G, AIF, Cyt c, Drp1, Bax, tBid, Bim, Bcl-X_L_, p53), mRNA (*PUMA*), Tissues (GSH, Caspase-3, Caspase-7), Histology	Chen, et al., 2019 [54]

**Figure 3:**
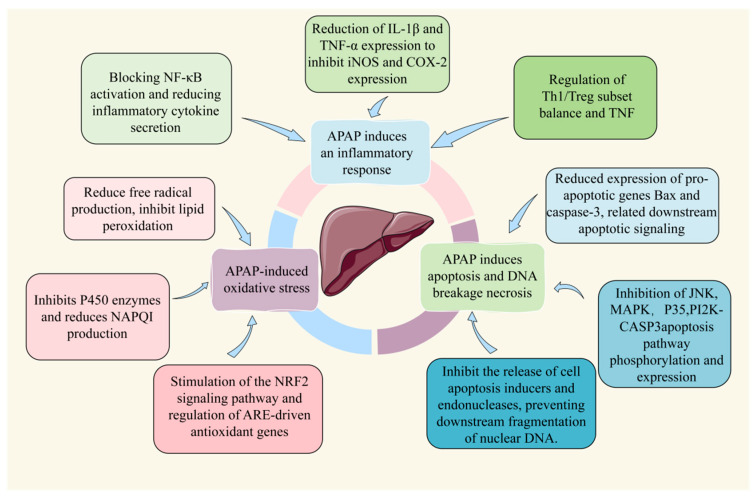
Summarizes the protective effects of phytochemicals against APAP-induced hepatotoxicity. This figure was partly generated using Servier Medical Art, provided by Servier, licensed under a Creative Commons Attribution 3.0 unported license.

DILI in pathological sections is characterized by liver edema, bleeding, slight liver fibrosis, and hepatocyte necrosis, which can help determine the presence and severity of DILI. Furthermore, scholars believe that measuring ROS, GSH, common proteins, mRNA, macrophages, and other factors associated with the mechanism of APAP-induced liver injury is useful and can be used to study the disease (Figure 3).

When studying the hepatotoxicity of APAP, mouse models have the advantages of short modelling times, low cost, significant effects, similar mechanisms to humans, and the ability to assess the degree of liver injury and potential hepatoprotective mechanisms in a relatively short period of time, which is helpful for clinical research. Therefore, male C57BL/6 mice are the most commonly used animal models.

### 3.2. In Vitro Models

Primary liver cells play a crucial role in the study of APAP hepatotoxicity. They provide assurance that they can simulate liver physiology, metabolism, and disease mechanisms. Obtained directly from organs, primary liver cells closely resemble the physiological conditions of the liver in vivo. They maintain important functional characteristics such as cell morphology, tissue structure, and metabolic activity, making them reliable representatives of natural liver cells [55,56]. By utilizing primary liver cells, researchers can evaluate the mechanism of APAP occurrence and gain key information for drug development and safety assessment. However, immortalized cells such as AML12, BRL 3A, and L-02 are still used for APAP research.

According to the literature search, the majority of cellular models (Table 2) are established based on animal experiments. Scholars typically isolate primary hepatocytes from mice, incubate them with 5–10 mM APAP, and measure cell viability and other indicators after 24 h. The study of cellular models is conducive to exploring specific mechanisms. Scholars have studied the specific mechanisms of APAP-induced liver injury and searched for new and more accurate evaluation criteria by measuring relevant mRNA and protein expression levels.

The APAP cell models provide us with an opportunity to study the intricate relationship between detection indicators and the biological phenomena of oxidative stress, inflammation, and apoptosis. ROS are caused by NAPQI. Initially, the cellular antioxidant defense system combats this oxidative stress by utilizing enzymes such as SOD. Moreover, liver damage is exacerbated by an inflammatory response mediated through the release of cytokines, such as TNF-α and IL-1β. These pro-inflammatory cytokines further perpetuate oxidative stress, worsening the condition. Additionally, key events involved in the progression of apoptosis within the APAP cell models include the release of cytochrome c, upregulation of caspases, and altered expression of Bcl-2 family proteins. By understanding these mechanisms, researchers can develop natural products to alleviate APAP-induced hepatotoxicity.

**Table 2:** Summary In Vitro Models.

Type	Dose of APAP	Pre Administration or Treatment	Administration Frequency	Medication Processing Time	Experimental Index	No. Reference
Primary hepatocytes	5 mM	Starve for 12 h	Once	For 6 h	Mitochondria (ROS, membrane potential), Cell quantification (TUNEL-positive primary hepatocytes, CD11b-positive cells, MPO-positive cells)	Yu, et al., 2023 [29]
Primary hepatocytes	10 mM		Once	For 12 h	Protein (NEDD4-1, VDAC1), LDH, HMGB1, mtROS	Zhu, et al., 2023 [31]
HepaRG cells	10 mM	Gene knockout	Once	For 12 h	Protein (Beclin-1, LC3I/LC3II, STING, IRF3, p-IRF3, IB: Flag, IB: HA, Myc-ST2, Flag, HA, HA-STING, Flag-TBK1, ATG5), mRNA (*IFN-β1, ISG54, ISG56*)	Wang, et al., 2023 [32]
AML-12 hepatocytes	10 mM	Incubated	Once	For 24 h	Protein (JNK, p-JNK, p-ATM, ATM, γH2AX, H2AX), ROS	Cen, et al., 2023 [33]
AML-12 hepatocytes and primary hepatocytes	5 mM	Incubated	Once	For 24 h	Cell viability, ROS, GSH, GSH/GSSG, mRNA (*Fdps*), Protein (GPX4, xCT, p-AKT, p-GSK3β, Nrf2), Fe^2+^	Zeng, et al., 2023 [34]
Hepatocytes	10 mM	Incubated	Once	For 6 h	Cell viability, ROS, Cell death evaluation, Protein (p-JAK2, JAK2, p-STAT3, STAT3, BAX, BCL-2),	He, et al., 2022 [36]
Primary hepatocytes	20 mM	Incubated	Once	-	Cell viability, Seahorse XFe96 metabolic flux, FFA, mRNA (*Acaa2*), Genome-wide analysis, Protein (CYP2E1), ND-1, FFAs, OCR, Luciferase activity assay (Acaa2 *N*-terminal promoter, truncated *N*-terminal promoters, WT, mutant *N*-2 promoters)	Lei, et al., 2022 [38]
AML-12 cells	10 mM	Incubated	Once	For 6 h		
Primary hepatocytes	10 mM	Incubated	Once	For 12 h	Protein (PRX3, NLRP3, GSDMD, Caspase-1, Cleaved Caspase-1, IL-1β, IL-18, PRX5, PRX6), Mitochondrial ROS, LDH	Wang, et al., 2021 [42]
AML-12 cells	5 mM	Transfection	Once	For 48 h	Cell viability, ROS, Cell proliferation, LDH, ROS, GSH, SOD, Protein (CCNA1, CCND1, CDK4, total NRF2, nuclear NRF2, pNRF2), mRNA (*Nrf2, Ho-1, Gstα, Gstμ*)	Zhou, et al., 2021 [44]
L-02 cells	10 mM	Incubate	Once	For 24 h	ROS, Protein (SIRT1, GPX4, NRF2, HO-1, Histone-H3)	Wang, et al., 2021 [45]
HepG2 cells	60 μM	Incubated	Once	-	Cell survival rate, Apoptosis rate, Serum Index (MAD, GSH, SOD), mRNA (*Bcl-2, Bax, Caspase-3*), Protein (Bcl-2, Bax, Caspase-3)	Zhao, et al., 2020 [57]
Huh7 cell	10 mM	Transfection	Once	For 24 h	Cell survival assay, Lysophosphatidylcholine, Protein (PRDX6), iPLA2, LPC	Lee, et al., 2020 [47]
AML-12 cells, primary hepatocytes	10 mM	Incubated	Once	For 4–8 h	Cell death determination, ROS, TNF-α, IL-1β, IL-6, Protein (p-JNK, JNK, Bcl-2, Bax, VDAC1, p-ASK1, ASK1, p-GSK3β, GSK3β, p-TRAF2, TRAF2, IκBα, p-IκB, p65, HDAC4, HDAC5, HDAC7)	Li, et al., 2020 [48]

## 4. Natural Product for APAP-Induced Acute Liver Injury

Natural product therapy is the use of natural products such as plant extracts, herbs, and traditional Chinese medicine formulations. It is primarily focused on utilizing natural compounds to prevent or treat APAP-induced liver injury, as many natural products have been reported to possess hepatoprotective effects. Plants and their derivatives have been part of active constituents due to the presence of bioactive components and they play an important role in the treatment and prevention of diseases. This systematic review focuses on select phytochemicals or plant extracts that have been shown to alleviate acute liver injury. There is a brief introduction, an overview of the proposed mechanisms, recent research findings, existing challenges, and prospects in combating APAP hepatotoxicity.

According to research conducted in the past five years, many herbal extracts inhibit inflammation, oxidative stress, apoptosis, and mitochondrial damage. These mechanisms can prevent or treat DILI caused by APAP to a certain extent. The only therapeutic drug approved for treating APAP-induced liver injury is *N*-acetylcysteine (NAC). The timely administration of NAC is crucial, although its efficacy is limited [4], and the oral bioavailability of supplemental formulations is low [58]. Since the discovery of APAP hepatotoxicity, animal models and cell experiments have been used to examine whether various phytochemicals or plant extracts possess hepatoprotective properties. Many experimental studies on phytochemicals or plant extracts have demonstrated hepatoprotective effects against APAP-induced liver toxicity models. Different types of herbal extracts may have similar hepatoprotective mechanisms.

### 4.1. The Physiological Function of Natural Products

#### 4.1.1. Oxidative Stress

In recent years, researchers have been particularly interested in antioxidant extracts from herbal products as hepatoprotective agents to reduce the toxicity caused by APAP. Natural products can help alleviate the inflammation and oxidative stress responses triggered by APAP-induced injury. In the model section, we have discussed changes not only in liver function but also in excessive inflammation and oxidative stress in APAP-induced liver injury. Natural compounds such as Salviae miltiorrhizae Radix et Rhizoma and Silybi fructus have shown reported anti-inflammatory and antioxidant effects.

Salviae miltiorrhizae Radix et Rhizoma, also known as Danshen, is a perennial upright herbaceous plant belonging to the Lamiaceae family and Salvia genus. The roots and rhizomes of Danshen are used as a medicinal herb for promoting blood circulation and removing blood stasis. Its components also possess cardiovascular protective, anti-inflammatory, and antioxidant effects. Two major bioactive compounds found in Danshen, liposoluble tanshinone and hydrosoluble salvianolic acid, inhibit APAP-induced liver toxicity through different mechanisms. It has been reported that salvianolic acid A (Sal A) [59], salvianolic acid B (Sal B) [60], salvianolic acid C (SAC) [61], and tanshinone IIA (Tan) [62] can counteract the elevation of AST and ALT induced by APAP. Sal A reduces APAP-induced liver toxicity by regulating miR-485-3p to increase SIRT12 expression in AML-12 cells [59]. In addition to its effects on inflammatory factors such as TNF-α, Sal A improves APAP-induced oxidative stress by modulating p66Shc and MnSOD [59]. Sal B upregulates Nrf1 via the PI3K and PKC pathways, thereby increasing HO-3 and GCLC expression and inhibiting APAP-induced liver injury [60]. SAC exhibits anti-inflammatory, antioxidant, and anti-apoptotic effects [61]. SAC reverses the increased expression of Keap1 and the decreased expression of Nrf2 and downstream target proteins induced by APAP, promoting Nrf2 nuclear translocation and activation of the Nrf2 pathway, exerting antioxidative effects [61]. Moreover, studies have shown that SAC has no effect on CYP2E1 expression in non-APAP-induced liver injury but can inhibit CYP2E1 expression under APAP-induced conditions [61]. CYP2E1 is a key enzyme in xenobiotic metabolism that triggers a series of events leading to APAP-induced liver toxicity. Inhibiting CYP2E1 expression can reduce APAP-induced liver toxicity to some extent, as seen in glycyrrhizin [63]. Tan promotes Nrf2 translocation into the nucleus, but its effects on Keap1 are not certain. It does not promote downstream HO-1 expression but increases the expression of Nrf2 target genes GCLC and NQO1, exerting antioxidative effects [62].


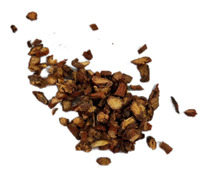


Silybi fructus, also known as shuifeili, is the mature fruit of the natural plant *Silybum marianum* (L.) Gaertn. It is a well-known herbal remedy widely used in liver diseases. The active component of Silybi fructus is a group of flavonolignans called silymarin, which includes silybin A/B, silychristin, and silydianin. Silymarin has been extensively studied for its hepatoprotective properties, meaning it helps protect the liver from damage and promotes its overall health and function. It primarily works by reducing the activity and expression of CYP2E1, as well as by decreasing the production of toxic metabolites, thereby preventing APAP-induced acute liver injury [64]. As an antioxidant and toxin blocker, silymarin reduces the generation of free radicals and lipid peroxidation, and the binding of toxins to hepatic cell membrane receptors. It also reduces or clears superoxide and peroxynitrite levels [65]. Furthermore, silymarin stimulates protein synthesis and liver regeneration, exhibiting anti-inflammatory and immune-modulatory activities [64]. Silybin A/B demonstrates its effects by upregulating the Nrf2/ARE pathway, reducing ROS levels, enhancing intracellular antioxidant enzyme activities, and inhibiting mitochondrial damage. It significantly improves the mitochondrial membrane potential changes in damaged hepatocytes, thereby preventing mitochondrial injury and the downstream activation of the Caspase-9/3 enzyme cascade. This, in turn, inhibits APAP-induced hepatocellular apoptosis and displays antioxidant effects against oxidative stress [66]. The above research findings suggest that silymarin has the potential to be further developed as an antioxidant in combating APAP-induced liver injury.


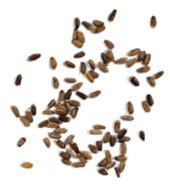


#### 4.1.2. Metabolic Pathways

Metabolic pathways are also important in the APAP model, and natural products can influence the drug-metabolizing enzymes involved in the metabolism of acetaminophen and its toxic metabolite, *N*-acetyl-p-benzoquinone imine (NAPQI). By modulating these enzymes, natural compounds such as Schisandra may potentially reduce the production of NAPQI, thereby limiting liver injury in cases of APAP overdose.

The main components of *Schisandra*, Schisandrin B (Sch B) and Schisandrin C (SC), have been found to exhibit protective effects on the liver. However, there are challenges in clinical applications due to their poor water solubility and low oral bioavailability. Sch B reduces the expression of ROS and inflammatory cytokines in liver cells, activates the pentose phosphate pathway, and inhibits the MAPK, JNK, and ERK signaling pathways, thus exerting antioxidant, anti-inflammatory, and anti-apoptotic effects [67]. SC decreases the expression of CYP2E1, inhibits cell apoptosis, improves inflammatory responses, and activates the Nrf2 signaling pathway, thereby inhibiting oxidative stress and alleviating APAP-induced liver injury [68]. Studies have shown that stem extracts of Schisandra significantly improve cell apoptosis, inflammation, and oxidative stress induced by APAP by modulating the MARK and Caspase-3 signaling pathways [69]. Additionally, it has been found that the activity of CYPs may be influenced by the duration of Schisandra administration. Short-term administration tends to inhibit CYP450 activity, while long-term administration may upregulate CYP activity. Currently, it appears that the protective effect of Schisandra against acetaminophen-induced liver toxicity is primarily dependent on the activation of the Nrf2 signaling pathway, with less influence on CYP450 activity [70].


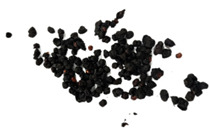


#### 4.1.3. Immunomodulation

Immunomodulation is a significant advantage of natural products, and it plays a vital role in our bodies. APAP-induced liver injury involves immune-mediated mechanisms that worsen liver damage. Natural products from herbs with immunomodulatory properties, such as ginseng or astragalus, may help regulate immune responses, reduce inflammation, and prevent further liver injury.

As for Ginseng, a traditional Chinese medicine, it is widely used for the treatment of various diseases and is among the most popular herbal remedies [71]. Ginsenosides, a class of steroidal compounds primarily isolated from the *Panax ginseng* C. A. Mey., have been identified as major active components [15,72]. Several ginsenosides have been discovered, with the primary bioactive constituents being Rk1, Rg1, Rg3, and others. These compounds possess pharmacological effects such as antioxidant, anti-apoptotic, anti-inflammatory, and anti-nitrosative actions. In animal experiments, they have demonstrated therapeutic effects against APAP-induced liver injury. However, there is a need for further docking studies in other binding sites as some positions show relatively weak docking forces. Experimental evidence has shown that ginsenosides can promote the expression of multidrug resistance proteins (MRPs) 1 and 3, upregulate Nrf2 and its target genes, inhibit the ERK and JNK MAPK pathways, reverse CYP2E1 overexpression, enhance antioxidant stress response, and exert hepatoprotective effects [73]. Additionally, they can influence NLRP3 inhibition, reducing the expression of iNOS and COX-2 in liver tissues and decreasing inflammation [74]. Furthermore, ginsenosides can activate the Nrf2/ARE pathway, lowering the protein expression level of Bax and the Bax/Bcl-2 ratio, leading to an anti-apoptotic state in cells [74]. Moreover, their anti-nitrosative effects can reverse the increased expression of 3-NT mediated by APAP [75]. Rg3, through AMPK-mediated autophagic flux, shows protective effects against sepsis-induced liver injury and mitochondrial dysfunction [76]. Therefore, ginsenosides primarily reduce the hepatotoxicity of APAP by inhibiting oxidative stress, inflammation, and apoptosis. From current perspectives, ginseng may be a promising candidate for the prevention and treatment of APAP-induced liver injury due to its multifaceted effects.


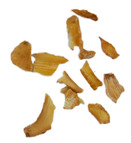


Scholars have been studying the mechanisms of compounds, extracts, and formulated extracts in relation to drug-induced liver injury (DILI). In addition to the aforementioned research, they have made discoveries regarding the hepatoprotective effects of various bioactive components present in natural products through various signaling pathways.

### 4.2. Compounds

**Figure 4:**
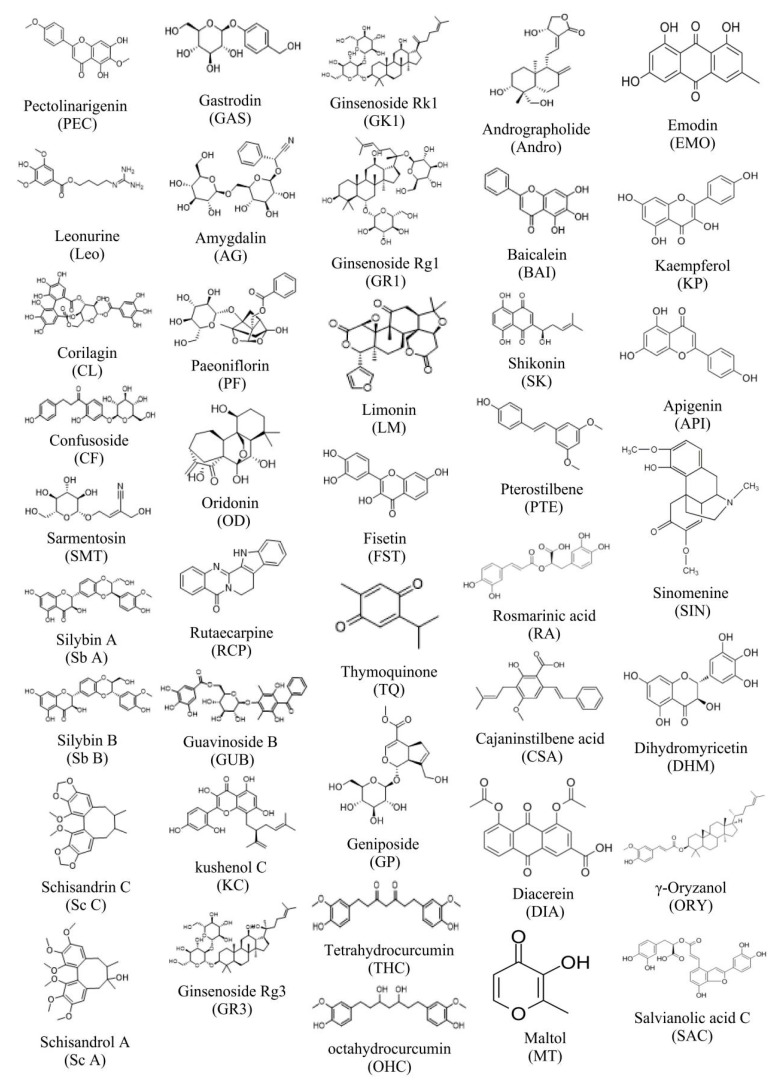
Structural formulae of phytochemical compounds and crude extracts used in the treatment of APAP-induced acute liver injury in past five years.

Natural product compounds are active ingredients with specific pharmacological effects found in natural products, and they possess a clear chemical molecular structure. In the study of the APAP model, these monomers are first utilized to explore their efficacy and mechanisms. This involves injecting compounds (Figure 4) into experimental animals to observe their effects on liver injury indicators and evaluate their efficacy and relevant research mechanisms. Additionally, compounds can also be evaluated for their impact on cells through in vitro experiments, such as cell culture experiments. These experiments can reveal the effects of monomers on aspects such as cell proliferation and inflammation, providing further understanding of their pathways in treating DILI; they may exert their effects by modulating various signaling pathways, including the Nrf2, PI3K/Akt, MAPK, and NF-κB signaling pathways (Table 3).

#### 4.2.1. Nrf2-Related Pathways

Compounds can activate the Nrf2 pathway, which, in turn, alleviates oxidative damage and provides protection against DILI. In cases of APAP-induced liver injury, the use of natural products has shown effective results in reducing cell damage caused by oxidative stress. These natural products achieve this by activating the Nrf2 signaling pathway, reducing the release of inflammatory factors, and regulating the synthesis, conjugation, and excretion of GSH. Nuclear factor erythroid 2-related factor 2 (Nrf2) is a transcription factor encoded by the NFE2L2 gene and can be indirectly activated by NAPQI [112]. Activated Nrf2 promotes the transcription of antioxidant enzymes, including quinone reductase 1 (NQO1), heme oxygenase-1 (HO-1), and microsomal epoxide hydrolase, and further stimulates GSH synthesis [113,114]. These Nrf2-activated antioxidant enzymes can act as defense mechanisms to detoxify NAPQI [113,114]. Fibroblast growth factor 21 (FGF21) induced by APAP overdose also increases the abundance of Nrf2 in the liver through peroxisome proliferator-activated receptor gamma coactivator-1 alpha (PGC-1α) expression [115]. This is a compensatory mechanism to protect against APAP hepatotoxicity, especially for curcumin and confusoside.

Curcumin (CMN) is a yellow polyphenol pigment and the main bioactive component found in the rhizomes of turmeric, commonly known as curcumin. Research data suggest that CMN exerts a protective effect against liver injury. Its protective effects are dose-dependent, with significant protection observed at higher doses [116]. Studies have demonstrated that CMN can reduce oxidative stress, inflammation, and cellular damage. In relation to oxidative stress, CMN hinders the activity of five P450 enzymes in a way that is dependent on its concentration [117], thereby reducing the production of NAPQI. It also inhibits Keap1, affects upstream mediators of Nrf2, regulates Nrf2 and its target gene expression, and promotes Nrf2 nuclear translocation, stimulating the Nrf2 signaling pathway and modulating antioxidant genes driven by ARE to protect cells against oxidative damage [118,119]. Additionally, studies have demonstrated that CMN suppresses APAP and causes apoptosis in liver cells by reducing the expression of the pro-apoptotic genes Bax and Caspase-3, inducing anti-apoptotic genes such as Bcl-x1, and increasing the Bcl2/Bax ratio [120]. CMN also exhibits functions such as restoring liver enzymes, inhibiting lipid peroxidation, preventing NF-κB activation, and reducing the secretion of inflammatory cytokines [121], thereby alleviating APAP-induced liver injury. Moreover, it can enhance the protective effects of NAC when used in combination, allowing for a reduction in the therapeutic dose of NAC [122]. The research findings indicate that CMN could be developed as an antioxidant against liver injury induced by APAP. However, the current challenge lies in its low oral bioavailability, which requires further exploration and solutions.


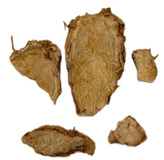


#### 4.2.2. NF-κB-Related Pathways

The activation of nuclear factor kappa B (NF-κB) is known to play a significant role in acute liver injury caused by excessive APAP [123]. Excessive APAP [124] can trigger the inflammatory response and activate NF-κB. Consequently, the activation leads to the release of various inflammatory cytokines, including TNF-α, IL-6, and IL-1β. These cytokines further exacerbate inflammation and contribute to liver damage [125]. However, certain natural product compounds, like limonin (LM) and paeoniflorin (PF), have the ability to inhibit inflammation and treat liver injury by suppressing the activation of the pathway.

Limonin (LM) is a bioactive compound derived from citrus plants and exhibits antioxidant activity [126]. It has been studied for its therapeutic effects on diseases such as LPS-induced liver injury and hepatic ischemia-reperfusion injury [127,128]. However, there is limited experimental research on its role in APAP-induced liver injury, and the mechanisms may not be fully understood. Based on existing studies, the effectiveness of limonin in alleviating APAP-induced hepatotoxicity is attributed to its activation of the Nrf2 antioxidant pathway and upregulation of Sirt1 to inhibit NF-κB-mediated inflammation. To some extent, limonin exhibits a similar ability to scavenge ROS as NAC. Additionally, treatment with limonin leads to a dose-dependent reduction in the Bax/Bcl-2 ratio and the inhibition of Caspase-3 cleavage, thereby alleviating mitochondrial dysfunction and apoptosis [97]. Therefore, limonin may be a promising and effective candidate for the treatment of liver injury.


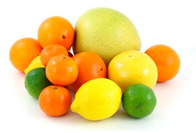


Paeoniflorin (PF), also known as paeoniflorin or peony glycoside, is the main active component derived from the lactate of Paeonia lactiflora. In traditional Chinese medicine, it has been used to treat various liver diseases [129]. Studies have demonstrated that PF exhibits a wide range of effects on the liver, protecting its anti-inflammatory, antioxidative stress, and anti-cell-apoptosis activities. It may alleviate bile stasis by inhibiting the activation of SIRT1/FXR and the NF-κB/NLRP3 inflammasome signaling pathway [130]. PF improves liver function indicators, tissue damage, and cell apoptosis activation caused by APAP by modulating CYP2E1, JNK, and their associated downstream cell apoptosis signals [88]. These findings broaden the application scope of paeoniflorin in liver diseases, indicating its potential role in the treatment of a drug-induced liver injury that warrants further exploration.


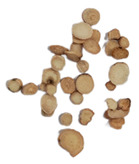


#### 4.2.3. MAPK-Related Pathways

The MAPK family is an important transmitter of signals from the cell surface to the nucleus [131]. It includes Erk1/2, Jnk1/2, and P38, which regulate cell proliferation and differentiation, cell stress, cell apoptosis, and other pathological processes [132]. The MAPK pathway that mediates inflammation and cell death plays a crucial role in liver injury. In the process of APAP-induced liver injury, inducing SIRT1 inhibits the P38 MAPK pathway, reduces the expression of apoptosis-related proteins such as Bcl-2 family proteins and Caspase family proteins, and inhibits cell apoptosis [125]. P38 also acts as a medium for cell survival in the cell cycle, increasing the expression and release of cell survival factors such as hepatocyte growth factor and insulin-like growth factor, promoting cell proliferation, and facilitating liver regeneration to alleviate acetaminophen-induced liver toxicity [125]. The MAPK pathway connects to the Nrf2 pathway, and inhibiting the MAPK pathway can induce Nrf2 activation and nuclear translocation, thereby enhancing the expression of downstream antioxidant genes, reducing the occurrence of cell stress responses, and ultimately reducing cell apoptosis [133,134,135]. When it comes to the treatment by compounds through the MAPK pathway, resveratrol (RE) and andrographolide (ADG) have regulate the MAPK pathway, thereby influencing the occurrence and severity of inflammatory responses. These molecules can intervene in key factors of the MAPK pathway, thereby modulating the inflammatory reactions associated with DILI. Overall, the improved text provides more details and clarifies the relationship between inflammatory responses and DILI, resulting in enhanced coherence and readability.

Resveratrol (RE) is a polyphenolic compound belonging to the stilbene family. It mainly derives from the dried rhizomes and roots of the Poygonaceae plant *Polygonum cuspidatum* Sieb. et Zucc., the skin and seeds of the fruit of the vine family *Vitis vinifera*, the seeds of the Fabaceae plant peanut Arachis hypogaea, and so on., exhibiting antioxidant properties in the dark but pro-oxidant properties under light conditions [136]. Increasing the expression of phase II and antioxidant enzymes has been shown to reduce oxidative stress in tissues. RE is an irreversible inhibitor of CYP3A4 and a non-competitive reversible inhibitor of CYP2E1. It also inhibits the activities of CYP3A11 and CYP1A2, preventing the activation of APAP to NAPQI [137]. Moreover, RE functions as a potent activator of SIRT1, promoting cell survival through the SIRT1-mediated deacetylation of p53 [138]. Additionally, it induces the expression of SIRT1, cyclin D1, cyclin-dependent kinase 4 (CDK4), and proliferating cell nuclear antigen (PCNA), facilitating liver regeneration and reducing hepatotoxicity induced by acetaminophen [137]. In therapeutic administration, RE reduces protein nitration by clearing peroxynitrite anions, inhibits the release of apoptotic inducers and endonucleases, and prevents downstream nuclear DNA fragmentation, thus protecting the liver [139]. Furthermore, studies have shown that RE significantly influences the Th1/Th2 cytokine balance, regulates the Th1/Treg subset balance, and affects TNF in APAP-induced liver injury [140,141]. RE may be an effective therapeutic option for APAP overdose. However, current research indicates that the bioavailability of RE metabolites in the intestine and liver is approximately 1%, and high-dose single ingestion may lead to adverse reactions [142]. Therefore, the relatively low bioavailability of RE in the body is still an issue to be addressed in clinical applications.


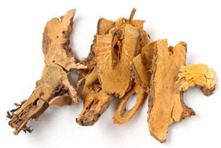


Andrographolide (ADG) is a compound found in Andrographis paniculata that belongs to the class of diterpenoids. This substance has diverse biological effects such as anti-inflammatory and liver-protective properties [143]. However, its poor solubility and cellular permeability have limited its clinical application [144]. ADG acts as a MAPK/Nrf2 pathway activator by inducing Nrf2 activation and its nuclear translocation, thereby enhancing the expression of downstream antioxidant genes to alleviate oxidative stress [109]. It binds to and antagonizes the function of PXR to inhibit CYP3A4 activity and reduce the formation of ROS [145,146]. ADG scavenges toxic free radicals, protecting mitochondria and organelles from ROS and nitrogen stress and cell death [147]. Experimental studies have shown that it exhibits more potent hepatoprotective effects than silymarin on liver cells [148]. Animal experiments have demonstrated that, when ADG is formulated into nanoparticulate hepatosomes, it exhibits higher bioavailability in liver tissue, rapidly restores antioxidant and glutathione levels, and protects the liver from acetaminophen (APAP) damage [149]. If the issues of poor solubility and cellular permeability can be addressed in the future, ADG could be a viable treatment option for those suffering from an APAP overdose.


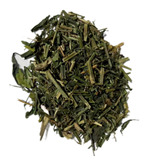


#### 4.2.4. PI3K/Akt-Related Pathways

The PI3K-Akt pathway is beneficial for liver regeneration following injury. Previous studies have indicated that activating the PI3K-Akt pathway can alleviate liver damage by inhibiting hepatocyte apoptosis and suppressing inflammatory pathways [124]. Upon activation, PI3K activates Akt, which, in turn, promotes GSK3β phosphorylation to achieve antioxidative stress effects [78,107]. The PI3K/Akt pathway can also facilitate the expression of Bcl-2 and inhibit the expression of Bax, thereby reducing APAP-induced liver necrosis resulting from an overdose [78]. Therefore, when investigating natural products for combating acute liver injury, the mechanisms related to the PI3K/Akt pathway should also be taken into consideration. Confusoside (CF) can also affect the connection between liver growth regulation and metabolism by regulating the PI3K/Akt-related pathways, promoting its internal balance.

Confusoside (CF) is a dihydrochalcone glucoside found in *Anneslea fragrans* Wall., a plant belonging to the Theaceae family and Anneslea genus. Widely distributed in southwestern China, this plant contains abundant biologically active molecules such as dihydrochalcone [81] in its leaves. The chemical structure of dihydrochalcone reveals its biological properties, including anti-inflammatory and antioxidant effects, which have significant implications in the prevention and treatment of liver injury [150]. CF, a dihydrochalcone glucoside, has been proven to prevent APAP-induced liver injury through three pathways [81]. Firstly, CF can interact with the Keap1–Nrf2 complex, leading to the release of Nrf2 and promoting its translocation into the cell nucleus [151]. The nuclear translocation of Nrf2 then stimulates the expression of downstream antioxidant enzymes such as SOD, CAT, NQO1, and HO-1, thereby reducing the levels of AST, ALT, and LDH in the serum and decreasing the MDA content in the liver tissue, exerting antioxidative effects [151]. Secondly, CF can inhibit the NF-κB pathway, thereby reducing factors such as TNF-α, IL-1β, IL-6, and NO, and exerting anti-inflammatory effects [81]. CF can also regulate the expression of pro-apoptotic factors such as Bcl-2 anti-apoptotic factors, Bax, and caspase-3/9 by activating the PI3K-CASP3 apoptotic pathway [152]. Therefore, CF can reverse the effects of APAP on oxidative stress and the Nrf2 pathway, thereby protecting liver cells. Currently, the precise mechanism of CF in apoptosis is not well understood. As a plant extract, further research on the biological properties of CF holds promise for its development as a novel therapeutic agent for liver injury.


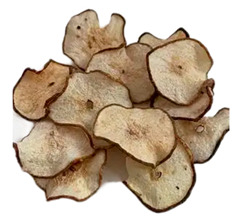


### 4.3. Natural Product Extracts

Natural product extracts are derived from natural resources such as plants, fruits, and herbs. These extracts are obtained through various methods, including soaking, distillation, or solvent extraction. By optimizing the use of solvents with different concentrations, it is possible to better separate the active compounds present in natural materials. The resulting extracts contain concentrated amounts of bioactive compounds that contribute to their antioxidant, anti-inflammatory, antimicrobial, and anticancer activities (Table 4). The main advantages of natural product extracts are their rich plant chemical composition. However, a distinct characteristic of these extracts, as opposed to individual compounds, is their complex chemical composition with many mixed components present.

Various natural extracts have anti-inflammatory, antioxidant, and anti-apoptotic properties. Currently, these plant extracts are being studied for their potential in mitigating APAP-induced liver damage. Mechanistically, plant extracts exert their effects by enhancing antioxidant defense, inhibiting metabolism, and CYP enzymes to reduce the formation of NAPQI, stabilizing cell membranes and protecting hepatocytes, promoting liver regeneration, reducing inflammatory cytokines, decreasing cell apoptosis, and reversing liver morphology caused by APAP toxicity. For example, as mentioned in the table, radish extract exhibits antioxidant and anti-apoptotic effects by increasing the expression of Nrf-2 and HO-1 and regulating the function of BAX and BCL-2, thereby reducing APAP-induced toxicity [153]. Methanol extract of P. Chaba stem bark enhances the antioxidant capacity, inhibits NAPQI formation mediated by hepatic toxicity, accelerates the release of cytokines from the liver, thereby increasing phase II enzyme levels, and expedites the harmless metabolism [154]. Here, we focus on introducing dandelion extract.

Dandelion (*Taraxacum officinale*) is a dietary medicinal plant and edible vegetable [155]. It contains abundant anti-inflammatory and antioxidant substances and has long been used in traditional medicine and folk remedies [156]. Based on the available experimental results [157], the dandelion extract may have preventive and therapeutic effects on APAP-induced acute liver injury through antioxidant, anti-inflammatory, and anti-apoptotic mechanisms: (1) Dandelion extract improves APAP-induced hepatotoxicity by increasing the expression of antioxidant proteins and Nrf2 [158]. High-dose dandelion extract has been shown to increase the expression of Nrf2 (the master regulator of antioxidant genes) in the liver, and the protein expression of Nrf2 target gene HO-1 in the liver is dose-dependent, leading to a better activation of the Nrf2 signaling pathway and suppression of oxidative stress [158]. (2) Dandelion extract inhibits the expression of iNOS and COX-2 by reducing IL-1β and TNF-α expression, thus improving APAP-induced hepatotoxicity. Therefore, the protective effects of dandelion extract may also be attributed to its anti-inflammatory activity [159]. (3) Dandelion extract suppresses the expression levels of Caspase-9 and inhibits JNK protein expression, thereby inhibiting oxidative stress and cell apoptosis through the suppression of the MAPK and NF-κB pathways, ultimately suppressing APAP-induced hepatocyte apoptosis [157]. Dandelion extract is expected to be a promising option for the treatment of APAP overdose in the future.


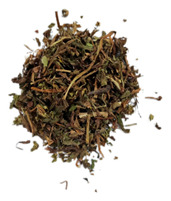


**Table 4:** Summarizes the natural product extracts used for the treatment of acute liver injury caused by APAP.

Phytochemicals	Source	Dose, Route, and Duration of Administration	Model	APAP Usage	Findings and Key Mechanisms	Reference
70% Ethanol Extract	Mulberry leaf, *Morus alba* L.	400 mg/kg, p.o. for 28 days	Male *Sprague–Dawley* rats	500 mg/kg, p.o.	Decreased CASP3, CYP2E1 immunoexpression, and antioxidant and anti-apoptotic properties.	Fadil, et al., 2023 [160]
70% Ethanol Extract	Olive leaf	400 mg/kg, p.o. for 28 days				
80% Methanolic Extract	A. Americana	100, 200, and 400 mg/kg, p.o. for 7 days	Male Wistar albino rats	2 mg/kg, p.o. single dose	Mechanism not explored.	Ayenew, et al., 2023 [161]
Aqueous Extract	Curcumin, *Curcuma aromatica* Salisb	1, 10, 25, 50, 100, 200, and 400 μg/mL for the Primary Cell	C57BL/6 mice Hepatocytes	2 mM	Increased the protein expression of Sirt1 and HO-1 vand increased the mRNA expression of these genes in liver tissue.	Kim, et al., 2023 [162]
		20, 100 mg/kg, i.p. once daily for 1 week	Male C57BL/6 mice	300 mg/kg, i.p. single dose		
80% Ethanol Extract	*Polygoni Multiflori* (Thunb.) Nakai, Polygoni Multiflori Radix	100, 200 mg/kg, p.o. for 21 days	Male Kunming mice	400 mg/kg, p.o. single dose	Upregulating antioxidant enzymes and repressing lipid peroxidation.	Wang, et al., 2023 [163]
Aqueous Extract	*Smilax china* L.	300, 600 mg/kg, p.o. for 14 days	Male BALB/c mice	400 mg/kg, p.o. single dose	Activated the Nrf2-ARE signaling pathway and inhibited oxidative stress.	Wang, et al., 2022 [164]
Petroleum and Ethanol Extract	*Broussonetia papyrifera*	100, 200, and 400 mg/kg, i.g. daily for 14 days	Male Kunming mice	200 mg/kg, i.g. single dose	Remodeling of intestinal flora, activation of Nrf2 pathway, inhibition of apoptosis.	Xu, et al., 2022 [165]
Aqueous Extract	*Amblygonocarpus andongensis*	125, 250, and 500 mg/kg, p.o. daily for 8 days	Male Wistar rats	1 g/kg, p.o. single dose	Inhibited oxidative stress and promoted oxygen radical scavenging.	Baponwa, et al., 2022 [166]
Ethanol Extract	Ficus exasperata	150, 250, and 500 mg/kg i.p. for 5 days	Albino rats	250 mg/kg, i.p. single dose	Mechanism not explored.	Adetuyi, et al., 2022 [167]
80% Methanol Extracts	*C. africana* Lam, Boraginaceae	100, 200, or 400 mg/kg, i.g. daily for ten days	Rats	2 g/kg, p.o. single dose	Anti-inflammatory and antioxidant activities of the plant.	Geresu, et al., 2022 [168]
Aqueous Extract						
Aqueous Extract	*Raphanus sativus* L.var niger, Radish	500, 1000 mg/kg, p.o. once daily for 4 weeks	Male BALB/c mice	500 mg/kg, i.p. single dose	Regulating antioxidant and anti-apoptotic signaling factors. Increased the expression of Nrf-2 and HO-1, and regulated the Bax and Bcl-2.	Hwang, et al., 2022 [153]
65% Methanol Extract	*Galium aparine* L. plants, Yogurt herb	Rats that drank tap water containing 250, 500 mg/kg for five days	Wistar albino rats	1500 mg/kg, p.o single dose	Improving the serum levels of liver enzymes and liver histology changes.	Sahin, et al., 2022 [169]
80% Aqueous Methanol Extract	*Melaleuca rugulosa* (Link) Craven	250, 500, and 1000 mg/kg, p.o. for 7 days, once a day	*Sprague–Dawley* male rats	3 g/kg, p.o. single dose	Oxidative stress-mediated activation of the JNK pathway in liver tissue.	Elsayed, et al., 2022 [170]
Aqueous Methanol Extract	*Paspalidium flavidum*, Watercrown grass	250 and 500 mg/kg, p.o. once a day for 7 days	Wistar rats (both male and female)	200 mg/kg, i.p. single dose,	hepatoprotective and gastroprotective, antioxidant properties.	Ismail, et al., 2022 [171]
20% Methanolic Extract	*Piper chaba* Hunter, Chui Jhal	250 and 500 mg/kg, p.o. once a day for 15 days	Male Sprague–Dawley rats	2 g/kg, p.o. single dose	Enhancing antioxidant defense and accelerating APAP harmless metabolism, inhibiting hepatotoxicity-mediated NAPQI formation.	Sarkar, et al., 2022 [154]
70% Methanolic Extract	Iris Florentina	250 and 500 mg/kg p.o. once a day for 7 days	Male albino rats	2 g/kg, p.o. single dose	Mechanism not explored.	Nawaz, et al., 2022 [172]
Aqueous Simmondsin-rich Extract	*Simmondsia chinensis*, Jojoba	0.6 mg/kg, p.o. until the 19th day	Male Swiss rats	2 g/kg, b.w	Inhibition of NF-kB pathway-based hepatocyte apoptosis and inflammatory stress.	Feki, et al., 2022 [173]
Simmondsin-Hydrolyzed Extract						
80% Methanol Extracts	*Taraxacum mongolicum* Hand.-Mazz, Dandelion	250, 500, 1000 g/120 mL, by oral gavage for 7 days	Kunming mice	350 mg/kg, i.p. single dose	Activating the Nrf-2/HO-1 pathway and inhibition of the intrinsic apoptosis pathway.	Zheng, et al., 2022 [159]
Root and Leaf Water Extracts		1 and 2 mM for Primary cells	Male SD rats	1 g/kg, p.o., once a day, five times a week for two weeks for rats	Activated the Nrf-2 pathway to reduce the level of oxidative stress. Inhibited pro-inflammatory cytokines. Attenuated the activation of the JNK pathway and reduced the expression of Bcl-2/Bax.	Wang, et al., 2022 [157]
80% Ethanol Extract		100, 200 and 400 mg/kg orally for 7 consecutive days	Kunming mice	350 mg/kg, i.p. single dose	Inhibited the occurrence of oxidative stress and apoptosis by suppressing MAPK and NF-κB pathways.	Ren, et al., 2020 [158]
0.5% Citric Acid in 80% Ethanol Extract	*Cleistocalyx nervosum* var. *paniala,* Ma-kiang	100 and 300 mg/kg, p.o. for 7 days, once a day.	Female Wistar rats	3000 mg/kg, p.o. single dose	Inducted antioxidant enzymes and detoxified enzymes leading to the restoration of GSH and a reduction in oxidative stress.	Chariyakornkul, et al., 2022 [174]
Acetone Extract	*Drynaria quercifolia* (L.)	1, 5 mg/kg, i.g. for 21 days, once a day.	Male Swiss albino mice	500 mg/kg, i.g. single dose	Inhibited the NF-κB-signaling pathway and activation of antioxidant response through increased expression of Nrf2.	Chatterjee, et al., 2022 [175]
70% Ethanol Extract	*Chrysanthemum morifolium* Ramat., Chrysanthemi Flos	110, 220 and 440 mg/kg, i.g. for eight days	Male Sprague–Dawley rats	800 mg/kg, i.g. single dose	Inhibit the excessive oxidative stress via GSK3β–Nrf2 pathway and reduce apoptosis through PI3K–Akt pathway.	Zhou, et al., 2021 [176]
Absolute Methanol Extract	*Pandanus odoratissimus* Linn., Pandanaceae	300, 600, 900 mg/kg, p.o. for 14 days.	Male Sprague–Dawley rats	3 g/kg, p.o. a single dose	Mechanism not explored.	Sinaga, et al., 2021 [177]
Aqueous extract	*Medicago denticulata*	100, 200, and 300 mg/kg, p.o. continued for 3 weeks	Rabbits	200 mg/kg, p.o. for 3 weeks	Lipid peroxidation.	Ahmad, et al., 2021 [178]
Ethanol Extract	*Orostachys fimbriata* (Turcz.) A. Berger	50, 100, or 200 mg/kg p.o. once a day for 7 days	Male C57BL/6 mice	300 mg/kg, i.p. single dose	Reduced CYPs via PXR inhibition and restoration of hepatic GSH content.	Zhou, et al., 2021 [179]
50% Ethanol Extract	*Centella asiatica* (L.) Urb., Centella Asiatica	50, 100, and 200 mg/kg/days, p.o. for 7 days	Male BALB/c strain mice	200 mg/kg, p.o. single dose	Antioxidant capacity and reduced expression of the inflammatory gene CYP2E1 transcripts.	Park, et al., 2021 [180]
Total Flavonoids	*Sedum sarmentosum* Bunge	50, 100, 200 mg/kg, no indication of the number of days of injection and method of administration	Male ICR mice	No concentration indicated	Inhibited the expression of Nrf2 and ARE proteins in the liver tissue.	Jiang, et al., 2021 [181]
Methanol Extract	*Acampe ochracea* (Lindl.) Hochr.	200, 100, and 50 mg/kg orally once daily for 14 consecutive days	Male Swiss albino rat	2 g/kg, oral, once	The VEGF signaling pathway to recover the injured hepatocytes.	Ahmed, et al., 2021 [182]
Aqueous extracts.	Date palm seeds; *Phoenix dactylifera* L.	200 and 400 mg/kg i.g. for six days	Male Wistar rats	1.5 g/kg, oral, once	Potent antioxidant capacity, membrane stabilizing effect and inhibit CYP, decreased the formation of NAPQI.	Bouhlali, et al., 2021 [183]
30% Ethanolic Extract	wheat, *Triticum aestivum* L.	100, 200 mg/kg, p.o. for six days	Male C57BL/6 mice	300 mg/kg, i.p. once	Mechanism not explored.	Lim, et al., 2021 [184]
Aqueous Extract	Dill shoots, *A. graveolens* L.	100, 200 mg/kg, p.o. daily for eight consecutive days	Male Wistar rats	2.5 g/kg, p.o. single dose, on the 9th day.	Increase in the level of GSH and TAC, and normalization of liver enzymes and inflammatory mediators.	Rasheed, et al., 2021 [185]
Triterpenoid-Enriched Extract	Guava Leaf, *Psidium guajava*	75, 150 mg/kg, p.o. daily for 7 days	Male C57BL/6 mice	300 mg/kg, i.p. once	The Nrf2 and MAPK signaling pathways.	Li, et al., 2021 [186]
		Compound 1 (1.1, 3.3, 10, and 30 μM), Compounds 6, 7, 16, and 17 (10 and 30 μM)	HepG2 cells	With 10 mM for another 48 h		
Hot-water Extract	Que Zui tea, *Vaccinium dunalianum* Wight	200 and 600 mg/kg, i.g. once daily for 7 consecutive days	Male Kunming mice	400 mg/kg, i.p. once	Enhance the expression of Nrf2, NQO1, and HO-1 proteins, suppressing the activation of MAPK signaling pathway. Enhancing the Bcl-2/Bax ratio and reducing caspase-3 and caspase-9 expressions in the liver tissues.	Wang, et al., 2021 [46]
Aqueous-ethanol Extract		200 and 600 mg/kg, i.g. once daily for 7 consecutive days				
Lipid-soluble (chloroform) Extract	*Dicranopteris linearis* (Brum.f.) Underw., Dicranopteris dichotoma	50, 250, or 500 mg/kg, p.o. for 7 successive days	Male SD rats	3 g/kg, p.o. single dose	Hepatoprotective activity, a high antioxidant capacity, improve the endogenous antioxidant enzymatic defense system.	Zakaria, et al., 2021 [187] Zakaria, et al., 2020 [188]
Licorice Extract	Dried roots, *Glycyrrhiza*	30 mg/kg, p.o. for 7 days	Male SD rats	500 mg/kg, i.p. single dose	Activated PI3K/Akt/Nrf2/HO-I, Keapl/Nrf2-ARE pathway and inhibited NF-κB/MAPK pathway.	Ma, et al., 2021 [189]
85% Ethanol Extract	Schisandra, *Schisandra* Michx.	10 mL/kg, i.g. one time each day for 7 days.	Male ICR mice	200 mg/kg, i.p. single dose	Attributed to the activation of ErbB signaling pathway and Arachidonic acid metabolism pathway, and inhibiting of oxidative stress, reducing ROS mediated DNA damage and inflammation, and reducing the metabolism of toxic substances of NAPQI.	Li, et al., 2021 [190]
50% Ethanol Extract		500 mg/kg, i.g. twice a day for three consecutive days.	Male C57BL/6J mice	400 mg/kg, i.p. single dose	Reducing intrahepatic diglyceride and triglyceride levels.	Yan, et al., 2020 [191]
Steam Shoot Extract	Ginseng, *Panax ginseng* C.A. Meyer	50, 100, 150, 200, and 250 mg/kg, i.g. daily for seven consecutive days	Grade ICR mice	250 mg/kg, i.p. single dose	Oxidative stress, reduced lipid peroxidation and inflammation, inhibited apoptosis.	Yao, et al., 2020 [192]
50% Ethanol Extract	*Isatis indigotica* Fort, Isatidis Folium	400 mg/kg, orally, for 7 consecutive days	Male ICR mice	100 mg/kg, i.v. once	Reducing the hepatic content of NAPQI, accelerating the generation of GSH and NAPQI-GSH adduct.	Ding, et al., 2020 [193]
Juice Extraction	Opuntia	800 mg/kg, p.o., single dose	Male Wistar rats	500 mg/kg, i.p. single dose	Scavenges ROS, increases antioxidant gene expression, and regulates the expression of important ros genes, decreases *Gadd45b* expression.	Gonzalez-Ponce, et al., 2020 [194]
		After 0.5 h, 10 mg/mL	Male Wistar rat primary hepatocytes	10, 20 mmol/L		
80% Ethanol Extract	*Vachellia nilotica* (L.) P. J. H. Hurter & Mabb, Acacia	100 mg/kg, i.g. daily for a month	Wister albino rats	750 mg/kg, i.g. daily for a month	Mechanism not explored.	Salman, et al., 2020 [195]
Stems, Leaves, and Roots Extract	*Leptadenia hastata,* Asclepiadaceae	250 mg/kg, orally, once daily for one week	Male and female albino mice	250 mg/kg, single dose	Antioxidant and anti-inflammatory activities.	Galani, et al., 2020 [196]
Ethanol Extract	aurantii fructus immaturus, *Citrus aurantium* L.	6 g/kg, i.g. daily for 7 consecutive days	Male SD rats	2 g/kg, p.o. single dose	Regulating lipid metabolism combined with conducting multi-targeted signaling pathway.	Shu, et al., 2020 [197]
Aqueous Extracts of Roots, Leaves, and Barks	Bixaceae, *Bixa orellana* L.	250 mg/kg, i.g. 3 h after paracetamol treatments once a day for one week	Albino mice	250 mg/kg, i.p. single dose,	Inhibited ERK signaling pathway, anti-inflammatory and antioxidant potential.	Gatsou Djibersou, et al., 2020 [198]
50% Aqueous Ethanol	Yellow Chinese Chive	25 and 100 mg/kg, p.o. daily for 7 days	Male ICR mice	700 mg/kg, i.p. single dose.	Activated the Nrf2 signaling pathway.	Kawakami, et al., 2020 [199]
Fold Boiled Water Extract	Sonneratia apetala	100, 200, and 400 mg/kg, i.g. per day for a week	Male Kunming mice	220 mg/kg, i.p. single dose	Scavenging activity against DPPH radicals, and has counteracted oxidative stress. Hepatic GSH levels and GSH-Px activity increased. Attenuating oxidative stress and increasing antioxidant enzyme activity. Attenuated the inflammatory response.	Liu, et al., 2019 [200]
70% Methanol Extract	*Myristica fragrans* Houtt., nutmeg	300 mg/kg, p.o. daily for a week	Male Wistar albino rats	2 g/kg, p.o. single dose	Enhance the antioxidant defence system, antioxidant, anti-inflammatory, and anti-apoptotic, promote the Nrf2/ARE pathway.	Dkhil, et al., 2019 [201]
70% Ethanol Extract	Nasturtium officinale	500 mg/kg, p.o. per day for a week	Male Wistar rats	2 g/kg, p.o. single dose	Increasing T-SH content as well as enhancing GPx activity. Antioxidant activity as a free radical scavenger.	Azarmehr, et al., 2019 [202]
Polyphenolic-rich Fraction Extract	*Lauridia tetragona* (L.f) R.H. Archer	500, 250, 125, and 62.5 μg/mL for 24 h	HepG2 cells	100 mM, single dose	Activated antioxidant enzyme synthesis through the Nrf2 pathway. Antioxidant activity and/or polyphenolic contents. Induced the expression of HO-1 and Nrf2 protein levels.	Odeyemi, et al., 2019 [203]
95% Ethanol Extract	*Veronica ciliata* Fisch.	900, 600, or 300 mg/kg, p.o. per day for two weeks	Male Kunming mice	150 mg/kg, i.p. single dose	Showed significant reversal by the decrease in hepatic functional enzyme activity, the inhibition of lipid peroxidation, and the increase in total serum antioxidant capacity (T-AOC) and antioxidant enzyme activity.	Lu, et al., 2019 [204]
		10, 25, and 100 μM, treated for 2 h	BRL-3A cells	20 mM, treated for 12 h	Involved in the p62-Keap1-Nrf2 signal pathway.	
Aqueous Extract	*Allium sativum* L., Garlic	25, 50, 100 mg/kg, i.g. for 7 days	Male Kunming mice	300 mg/kg, i.p. single dose	Inhibited CYP2E1, and activated Nrf2 pathway.	Zhao, et al., 2019 [205]

### 4.4. Formula Extract

Formula extract is a concentrated mixture or solution extracted from various medicinal herbs or natural ingredients. It is typically obtained through a specific formulation process designed to extract and combine specific components or compounds from the source material. These formulations can be used to treat APAP-induced liver toxicity, as shown in the table that demonstrates their protective effects (Table 5). The protective effects are mainly attributed to their antioxidant and membrane-stabilizing activities.

The combination of multiple herbal ingredients from pomegranate pine, Roxburgh pine, Chirata pine, butterfly orchid, and Amorphophallus konjac in these formulations can effectively target multiple pathways and improve bioavailability. Mechanistically, the formulations exert antioxidant effects by eliminating excessive free radicals and inhibiting lipid peroxidation, which contributes to stabilizing cell membranes and reducing APAP-induced liver damage. However, the exact interactions between these ingredients require further investigation and examination [206].

Pien Tze Huang, a nationally protected formula, is currently known to consist of 3% musk, 5% cow-bezoar, 85% gastrodia, and 7% snake gall. It has a long history of medicinal use. The mechanism by which this formation treats APAP-induced liver injury involves increasing autophagy and inhibiting the NLRP3 inflammasome, and the effect is inhibited by 3-MA [207]. Further research is needed to identify and evaluate the presence of bioactive components responsible for the hepatoprotective effect in Pien Tze Huang and determine if these components are present in other plants, thereby expanding the range of plant extracts or herbal formulations that can be used for treatments.

However, compound formulations are characterized by having multiple targets and mechanisms due to the combination of known active ingredients. Although there has been limited research on the efficacy and safety of global formulae in treating APAP-induced liver injury, these formulations show promising prospects for future development.

**Table 5:** Summarizes the formula extract used for the treatment of APAP-induced acute liver injury.

Formula Extract	Dose, Route, and Duration of Administration	Animals	APAP Dose and Route of Administration	Findings and Key Mechanisms	Reference
GTS-LE	55.00 mg/kg Sal B, 8.60 mg/kg TSN II_A_, 33.13 mg/kg GA, i.g.	Male Kunming mice	500 mg/kg, i.g. single dose	Inhibited oxidative stress and lipid peroxidation.	Zhang, et al., 2022 [208]
Pien Tze Huang	75, 150, or 300 mg/kg, 2 times/day for 3 days, p.o	WT- C57BL/6 mice	400 mg/kg, i.p. single dose	Inhibition the NF-κB pathway, NLRP3 inflammasome, and promoted autophagy.	Zhao, et al., 2023 [207]
HF	100, 200, and 400 mg/kg, p.o. for 15 days	Either sex wistar rats	640 mg/kg, p.o. single dose	Hepatoprotective and antioxidant.	Kataki, et al., 2022 [209]
LWWL	Schisandrin A or Schisandrin B 10, 20, and 40 μM	HepaG2 cells	20 mM for 12 h	Inhibited the NF-κB signaling pathway and suppressed H2O2-induced cell apoptosis, and inhibited the release of ROS.	Gao, et al., 2021 [210]
	159.78 mg/kg Schisandrin A, 162.43 mg/kg Schisandrin B, 0.93 mg/kg esculetin, 8.56 mg/kg luteolin	Male SD rats	300 mg/kg, i.p. single dose		
Gamisoyo-san	100–400 μg/mL for 2 h	BNL CL.2 cell	20 mM, single dose	Lipid peroxidation.	Jin, et al., 2021 [211]
	700 mg/kg, 1400 mg/kg, p.o. for 7 days	Male BALB/c mice	200 mg/kg, i.p. single dose		
A multi-herbal combination (MHC)	50, 100, and 200 mg/kg, i.g. once daily for seven consecutive days	Female Wistar rats	3 g/kg, i.g. single dose	Triggering antioxidant defense systems, and stabilizing cell membranes by inhibiting LPO.	Kaur, et al., 2021 [206]
Yan-Gan-Wan (YGW)	Pre or post:100 mg/kg, i.g. once daily for 7 or 14 days	Male ICR mice	400 mg/kg, i.p. single dose	Reducing collagen fiber formation, mitigates oxidative stress, inflammatory factors, and apoptosis, and inhibits the expression of TNF-α and caspase-3.	Yeh, et al., 2021 [212]

## 5. Discussion and Prospects

The incidence of drug-induced acute liver injury is increasingly common and has gradually become a significant cause of liver failure. This increase in drug-induced liver injury cases has significant implications for public health and requires a thorough understanding of the mechanisms behind these injuries. One such drug that frequently leads to acute liver injury is APAP, a commonly used antipyretic and analgesic drug. However, its improper use can result in severe consequences, making it important to delve into the mechanisms underlying APAP-induced liver injury.

The most common model used to study it is the mouse model, which offers several advantages for research purposes. Male mice are particularly favored in APAP studies due to their more pronounced modeling effects at lower doses than female mice. Typically, a dose of 300 mg/kg is sufficient to achieve a successful model. However, during the modeling process, dissolving APAP in common solvents poses challenges, leading experimenters to resort to alternative methods such as dissolving APAP in warm saline and heating the reagent above 60 degrees Celsius. It is necessary to consider whether this change in temperature affects the modeling of acute liver injury. To ensure accurate modeling, it is crucial to exclude the potential impact of temperatures on injury during the experimental process.

For cellular models, researchers tend to isolate primary liver cells, as they closely reflect the physiological characteristics of the liver. In these models, doses of 5–10 mM APAP are typically considered adequate for inducing liver injury. These cellular models provide valuable insights into the mechanisms underlying APAP-induced liver injury and can contribute to the development of therapeutic strategies.

The success of models in studying APAP is an often overlooked but critical issue in experiments. We found that most scholars judge the adequacy of modeling by examining pathological sections of male mouse livers and serum levels of ALT and AST, while only a small portion of scholars examine indicators in plasma. Mouse blood collection is often performed using the retro-orbital sinus, and, to obtain plasma after centrifugation, heparin often needs to be added to the collection tubes before centrifugation due to the minimal volume of blood collected. Therefore, we deemed serum indicators more appropriate. Additionally, we can evaluate the modeling and therapeutic effects by observing pathological tissue sections. Pathological conditions can reflect the severity of liver injury. Most scholars use classic HE staining, immunohistochemistry, and immunofluorescence analysis, while some use TUNEL staining to observe liver apoptosis and Masson staining to observe liver fibrosis.

**Figure 5:**
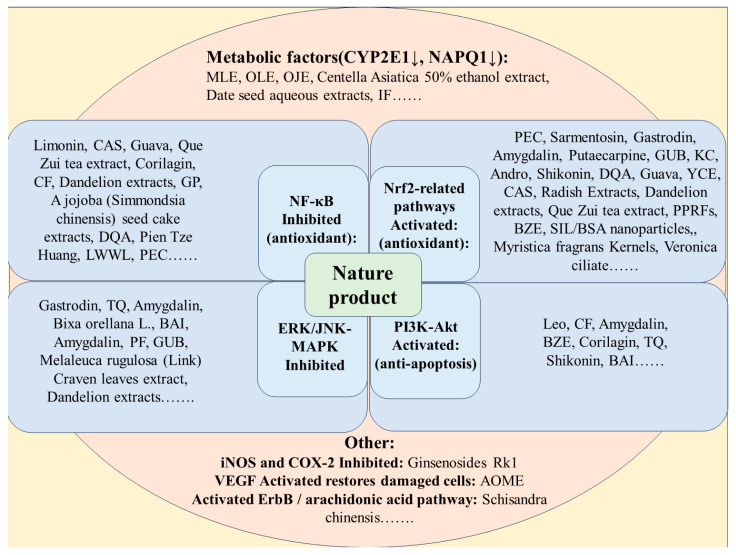
Summarizes the protective effects of phytochemicals against APAP-induced hepatotoxicity.

Understanding the pathogenesis of APAP-induced acute liver injury is crucial for developing effective therapeutic strategies. The pathogenesis involves multiple factors, including oxidative stress, inflammation, and cell apoptosis. Oxidative stress can be assessed by detecting ROS in liver tissues. Inflammation can be examined by measuring inflammatory factors such as TNF-α, IL-6, and IL-1β. Cell apoptosis, another critical factor in liver injury, can be evaluated by examining the expression of the pro-apoptotic factor Bax and the anti-apoptotic factor Bcl-2. Researchers are also exploring new indicators, such as cfDNA, for prediction and monitoring purposes. Additionally, the involvement of specific pathways, like the STING pathway and the JNK pathway, is being investigated to understand the underlying mechanisms. Different types of herbal extracts may have similar hepatoprotective mechanisms. For example, CAS [162], radish extracts [153], dandelion extracts [159], Que Zui tea extract [46], PPRFs [203], and so on can all inhibit liver inflammation by activating the Nrf/HO-1 pathway. Different extracts from the same plants can activate different pathways and exert their respective effects. For instance, Ginsenosides Rg3 regulates the NlRP3 pathway and inhibits inflammation [76]. Ginsenoside Rk1 can inhibit iNOs and COX-2, and promote anti-apoptosis (by inhibiting Bax and increasing Bcl-2) [74]. Ginsenoside Rg1 activates the Nrf2/ARE pathway to counteract oxidative stress and suppresses caspase-8/9 to prevent apoptosis [96] (Figure 5).

Understanding disease pathogenesis opens up a new research direction. Currently, there are no effective drugs for drug-induced liver injury. However, we have found that traditional Chinese herbs and their chemical extracts exert certain therapeutic effects and possess the advantages of fewer side effects, lower toxicity, and lower costs than synthetic drugs. We reviewed the treatment strategies of plant-derived chemical substances for this type of drug-induced liver injury. For example, silymarin, curcumin, and berberine have shown promising results in in vivo and in vitro studies in mice, indicating their ability to alleviate APAP-induced liver toxicity through mechanisms such as reducing oxidative stress and inflammation. However, the specific mechanisms of these plant-derived chemical substances still require examination, and, to achieve their therapeutic outcomes, further preclinical trials and clinical studies are necessary. Although many plant extracts have been reported to exert therapeutic effects against preclinical drug-induced liver injury, their application in clinical settings remains a significant task.

Furthermore, there are challenges in the development of these plant-derived chemical substances as therapeutic drugs, such as the poor solubility, incomplete understanding of specific mechanisms, low bioavailability, and incomplete understanding of their effects on the human body. Future research should focus on elucidating precise molecular targets and signalling pathways, improving the bioavailability of drugs, enhancing therapeutic efficacy, and reducing side effects. Additionally, the exploration of combination therapy and drug delivery systems should be conducted to mediate the effects of drugs in an efficient and low-risk manner.

In conclusion, plant-derived chemical substances such as silymarin, curcumin, and berberine hold potential in the treatment of drug-induced liver injury caused by APAP overdose. Further research on the specific mechanisms of these chemical substances, risk assessment, and their application in clinical trials is crucial for the translation of these substances into frontline drugs for APAP-induced disease.

Therefore, natural products can be utilized preventively to protect the liver from severe damage, making them a valuable treasure trove of medicines. Their potential is worth exploring and developing. Future research on new drugs derived from natural products will become one of the key methods for treating AILI, as the various components of natural medicines provide multiple targets for disease treatment.

## Figures and Tables

**Table 3:** Summarizes the phytochemical monomers and crude extracts used for the treatment of acute liver injury caused by APAP.

Phytochemicals	Dose, Route, and Duration of Administration	Model	APAP Dose and Route of Administration	Findings and Key Mechanisms	Reference
Pectolinarigenin (PEC)	5 and 10 mg/kg daily, i.p. for 7 days	Male C6BL/8 mice	400 mg/kg, i.p. single dose, on 7th day after the final dose 2 h	Activate Nrf2 and PPARα signaling, decreasing hepatic oxidative stress and inflammation, increasing phase IIdetoxification enzymes related to APAP metabolism.	Li, et al., 2023 [77]
	0, 0.25, 0.5, 1, 2, and 5 μM for 48 h before APAP administration	HepG2 cells	0, 2.5, 5, 10, 20, and 40 mM for 6, 12, and 24 h.		
Leonurine (Leo)	20 and 40 mg/kg i.g. for 7 days	Male ICR mice	300 mg/kg, i.p. single dose	Alleviated ALI by modulating the PI3K/AKT signaling pathway.	Yu, et al., 2023 [78]
	10 μΜ Leo for 12 h	Mouse primary hepatocytes	DMEM with 10 mM APAP for 24 h		
Corilagin (CL)	0, 1, 5, or 10 mg/kg i.p. after APAP administration	Male C57BL/6C mice	300 mg/kg, i.p. single dose	Involve the regulation of IL-6/STAT3 and MAPK/NF-κB pathways through NOX-derived ROS.	Liu, et al., 2023 [79]
	15, 30, 60 mg/kg i.p. twice for one day	Male C57BL/6 mice	400, 900 mg/kg, i.p. single dose	Inhibited AMPK, upregulated GSK3β, and activated the Nrf2 pathway.	Lv, et al., 2019 [80]
	7.5, 15, or 30 μM, treated for 25 h	HepG2 cells	15 mM, treated for 24 h		
Confusoside (CF)	50, 100 μg/mL	Hepatoma HepG2 cells	10 mM	Inhibiting the NF-κB-regulated inflammatory response and PI3K/Akt-regulated apoptosis.	Zhao, et al., 2023 [81]
Rosmarinic acid (RA)	20, 40, or 80 mg/kg, Unknown route of administration, for seven days	Male Kunming mice	500 mg/kg, i.p. single dose	Upregulated Nrf2 pathway and inhibited NEK7-NLRP3 pathway.	Yao, et al., 2022 [82]
	20, 40, or 80 μM	HepG2 cells	25 mM		
Cajaninstilbene acid (CSA)	75 mg/kg, p.o. after APAP administration	Male C57BL/6 mice	300 mg/kg, i.p. single dose	Activated Sestrin2-LKB1-AMPK pathway, enhanced mitochondrial quality control, and inhibited oxidative stress.	Yan, et al., 2022 [83]
	50 μM	Mouse primary hepatocytes	5 and 10 mM		
Sarmentosin (SMT)	0.5, 1, 2 μM to L-02 and 5, 10, 20 μM to AML-12 cells for 2 h	L-02 and AML-12 cells	20 mM for 2 h single dose	Regulating Nrf2 to alleviate APAP-induced hepatocytes oxidative damage.	Jiang, et al., 2022 [84]
	20, 40, and 80 mg/kg for a week	Male ICR mice	300 mg/kg i.p. single dose		
Silybin A/B (SB A/B)	20 mg/kg, single dose, 30 min after APAP administration.	BALB/c mice	300 mg/kg, i.p. single dose	Regulated the Nrf2/ARE pathway, inhibited mitochondrial damage and apoptosis pathway associated with caspase-9/3.	Ding, et al., 2022 [66]
Schisandrin C (Sc C)	200 mg/kg, i.g. for 7 days	Male C57BL/6 mice	400 mg/kg, i.p. single dose	Regulated the Nrf2 signaling pathway and reduced the expression of inflammatory factors and CYP2E1.	Dai, et al., 2022 [68]
Schisandrol A (Sc A)	10 g/kg, i.g. one time each day for 7 days.	Male ICR mice	200 mg/kg, i.p. single dose, after the last intragastric administration for 1 h	Activated TNF signaling pathway, and inhibited the activities of cytochrome P450 enzymes.	Li, et al., 2022 [85]
Gastrodin (GAS)	0, 15, 30, or 45 mg/kg, i.p. single dose, at 30 min after APAP administration.	Adult C57BL/6 (B6) mice	300 mg/kg, i.p. single dose	Inhibited ERK/JNK MAPK signaling pathway while activating Nrf2 signaling pathway	Liao, et al., 2022 [86]
Amygdalin (AG)	2.5, 5 mg/kg i.p. at the same time	Male C57BL/6 mice	400 mg/kg, i.p. after fasting for 15–17 h; the mice were intraperitoneally injected.	Inhibit apoptosis and necrosis, alleviate inflammation and oxidative stress, and even reverse liver injury by activating the AKT/JNK/Nrf2 pathway.	Zhang, et al., 2022 [87]
Paeoniflorin (PF)	60 mg/kg, i.g., for 5 days, once a day.	Male C57BL/6 mice	250 mg/kg, i.p. single dose	Regulated CYP2E1/JNK signaling.	Deng, et al., 2022 [88]
Diacerein (DIA)	25 and 50 mg/kg, p.o. for 7 days	Male albino rats	1 g/kg, p.o. single dose	Reduced oxidative stress, anti-inflammatory, anti-apoptotic, elevated PPAR-γ expression, and inhibited HMGB-1/TLR4/NF-κB signaling pathway.	Mohamed Kamel, et al., 2022 [89]
Emodin (EMO)	15 and 30 mg/kg, p.o. for 5 consecutive days.	Male C57BL/6 mice	300 mg/kg, i.p. single dose	Inhibited cCGAS-STING pathways.	Shen, et al., 2022 [90]
Oridonin (OD)	20 mg/kg, i.p. after APAP administration 1 h	Male C57BL/6 mice	300 mg/kg, i.g. single dose	Targeted the B. vulgatus–urea cycle–Nrf2 axis and enriched the gut microbiota regulation of B. vulgatus.	Hong, et al., 2021 [91]
Rutaecarpine (RCP)	5 or 20 mg/kg once daily for 7 days	Male ICR mice	300 mg/kg, i.p. single dose	Inhibited CYP2E1 expression, inhibited lipid peroxidation, and activation of Nrf2 pathway.	Choi, et al., 2021 [92]
Guavinoside B (GUB)	3.3, 10, 30, and 100 μM	HepG2 cells	5 mM.	Activated the Nrf2 pathway, and inhibited the JNK pathway.	Li, et al., 2020 [93]
	100 mg/kg/day, i.g. for 7 consecutive days	Male C57BL/6 mice	300 mg/kg, i.p. single dose, after the final administration		
kushenol C (KC)	1, 10, 20 mg/kg, p.o. every day for seven days.	Male BALB/c mice	500 mg/kg, i.p. single dose, after the final administration	Regulated Caspase 3, Bax/Bcl-2, GSH, and ROS production and activated the AKT-mediated Nrf2 pathway.	Cho, et al., 2021 [94]
	Various concentrations for 1 h	HepG2 cells	1 mM tBHP, for 24 h		
Kaempferol (KP)	250 mg/kg, p.o. for 7 days	Male Wistar rats	800 mg/kg, i.p. single dose	Activated SIRT1, inhibited PARP1 and CYP2E1.	BinMowyna, et al., 2021 [95]
Ginsenoside Rg3 (GR3)	5, 10, and 20 mg/kg, p.o. after administration APAP	Male C57BL/6J mice	350 mg/kg, p.o. single dose	Regulated NLRP3 pathway.	Gao, et al., 2021 [76]
Ginsenoside Rk1 (GK1)	10 mg/kg, 20 mg/kg, i.g. per day for a week	Male ICR mice	250 mg/kg, i.p. single dose	Inhibited the increase of iNOS and COX-2 expression. Regulated Bax and Bcl-2.	Hu, et al., 2019 [74]
Ginsenoside Rg1 (GR1)	Treated for 24 h	HepG2 and HEK293 cells	16 mM for 24 h	Activated Nrf2/ARE signaling pathway. Decreased oxidative stress. Inhibited ROS. Decreased the activation of the downstream caspase-8/9 to inhibit apoptosis of cells.	Gao, et al., 2019 [96]
	No treatment	Male C57BL/6 mice	500 mg/kg, i.g. single dose		
Limonin (LM)	10, 25, 50 μM, for 2 h	L-02 cells	7.5 mM, treated for 24 h	Upregulated Sirt1, inhibited NF-κB inflammatory response, and activated Nrf2 antioxidant signaling.	Yang, et al., 2020 [97]
	40, 80 mg/kg, p.o. for 1 h	Male C57BL/6 mice	300 mg/kg, i.p. single dose		
Apigenin (API)	20 and 80 mg/kg for 7 consecutive days.	Male C57BL/6 mice	400 mg/kg, p.o. single dose	Interacted with SIRT1 and activated autophagy.	Zhao, et al., 2020 [98]
	5, 50 μM	L-02 cells	10 mM, for 48 h		
Sinomenine (SIN)	25, 50, and 100 mg/kg, unknown route of administration, for 7 days	Male C57BL/6 mice	250 mg/kg, i.p. single dose	Inhibit the activation of NLRP3. Activated TGF-B/Smad pathway.	Chen, et al., 2020 [99]
	10, 50, and 100 μg/mL for 24 h.	BRL-3A cell	75 mM, single dose		
Dihydromyricetin (DHM)	25, 100, 200 mg/kg, i.g. daily for 5 days	Male C57BL/6 mice	300 mg/kg, i.p. single dose	Inhibited hepatocyte death, promoted regeneration, and regulated the lipid homeostasis imbalance mediated by PPARs and SREBP-1c.	Dong, et al., 2019 [100]
γ-Oryzanol (ORY)	5 and 10 μg/mL for 24 h	L-02 cells	15 mM	Inhibited NF-κB p65 pathway, inhibited AMPK, upregulated GSK3β, and activated the Nrf2 pathway.	Shu, et al., 2019 [101]
	7 and 14 mg/kg, i.g. for 7 days	Male Kunming mice	300 mg/kg, i.p. single dose		
Fisetin (FST)	10, 20, and 40 mg/kg, i.g. for 7 days.	Male C57BL/6 mice	400 mg/kg, i.p. single dose	Induced the increasing of glutathione metabolism and enhanced expression of downstream antioxidative enzymes.	Zhao, et al., 2019 [102]
	Treated with FST for 15 min	L-02 cells	Incubated with APAP for 24 h		
Thymoquinone (TQ)	5 or 20 mg/kg, i.p. single dose at 2 h before the administration of APAP	Male Kunming mice	300 mg/kg, i.p. single dose	Inhibited MAPK and JNK phosphorylation, and activated the AMPK signaling pathway to inhibit the PI3K/mTOR signaling pathways.	Cui, et al. 2019 [103]
Geniposide (GP)	10, 30, and 100 mg/kg, p.o. for three times at 24 h before the injection of APAP	Male C57BL/6 mice	350 mg/kg, i.p. single dose	Downregulated CYP2E1 expression, ameliorate oxidative stress. Suppressed the TLR4/NF-κB signaling pathway.	Yang, et al., 2019 [104]
Tetrahydrocurcu-min (THC)	25, 50, and 100 mg/kg, i.p. single dose at 30 min before the administration of APAP	Male Kunming mice	220 mg/kg, i.p. single dose	Inhibited CYP2E1 activity and activated the Nrf2 pathway.	Luo, et al., 2019 [105]
Octahydrocurcumin (OHC)					
Pterostilbene (PTE)	15, 30, and 60 mg/kg, i.p., single dose at 1 h after the administration	Male ICR mice	400 mg/kg, i.p. single dose	Restoring impaired autophagic flux.	Kang, et al., 2019 [106]
Shikonin (SK)	12.5, 25.0 mg/kg, i.p., single dose at 1 h before the injection of APAP	Male C57BL/6 mice	500 mg/Kg, i.p. single dose	Inhibited oxidative stress through AKT/GSK3β pathway-dependent Nrf2 upregulation.	Li, et al., 2018 [107]
	0.25, 0.375, and 0.50 μM for 24 h	L-02 cells	10 mM, for 24 h		
	12.5 mg/kg, i.p. for 2 h	Male BALB/c mice	300 mg/kg, i.p. single dose	Inhibited the expression of TLR9 and NLRP3, and suppressed oxidative stress.	Guo, et al., 2019 [108]
	5 μM	AML-12 cells	0, 5, 10, 20, 50, 100 mM for 24 h		
Andrographolide (Andro)	20, 40 mg/kg, i.g. for 4 weeks	C57BL/6 mice	300 mg/kg, i.g. single dose	Induced Nrf2 activation and attenuating hepatic oxidative stress injury.	Yan, et al., 2018 [109]
Baicalein (BAI)	100, 50 mg/kg, p.o. per day for a week	Kunming male mice	350 mg/kg, i.p. single dose	Related to the regulation of AKT-mTOR, MAPK, and JAK2/STAT3 signaling pathways.	Zhou, et al., 2018 [110]
Salvianolic acid C (SAC)	5 mg/kg, 10 mg/kg, and 20 mg/kg, i.p. daily for 6 days	Male ICR mice	400 mg/kg, i.p. single dose	Activated Nrf2 pathway, inhibited MAPK and NF-κB pathway, inhibited apoptosis and inflammation.	Wu, et al., 2019 [61]
Maltol (MT)	50 and 100 mg/kg, i.p. for 7 days	Male ICR mice	250 mg/kg, i.p. single dose	Inhibited oxidative-stress-mediated NF-κB pathway activation and apoptosis, and activated PI3K/Akt pathway.	Wang, et al., 2019 [111]

## Data Availability

Not applicable.
